# How many sightings to model rare marine species distributions

**DOI:** 10.1371/journal.pone.0193231

**Published:** 2018-03-12

**Authors:** Auriane Virgili, Matthieu Authier, Pascal Monestiez, Vincent Ridoux

**Affiliations:** 1 Centre d’Etudes Biologiques de Chizé - La Rochelle, UMR 7372 CNRS—Université de La Rochelle, Institut du Littoral et de l’Environnement, La Rochelle, France; 2 Observatoire PELAGIS, UMS 3462 CNRS—Université de La Rochelle, Systèmes d’Observation pour la Conservation des Mammifères et des Oiseaux Marins, La Rochelle, France; 3 BioSP, INRA, Avignon, France; Sanya Institute of Deep-sea Science and Engineering Chinese Academy of Sciences, CHINA

## Abstract

Despite large efforts, datasets with few sightings are often available for rare species of marine megafauna that typically live at low densities. This paucity of data makes modelling the habitat of these taxa particularly challenging. We tested the predictive performance of different types of species distribution models fitted to decreasing numbers of sightings. Generalised additive models (GAMs) with three different residual distributions and the presence only model MaxEnt were tested on two megafauna case studies differing in both the number of sightings and ecological niches. From a dolphin (277 sightings) and an auk (1,455 sightings) datasets, we simulated rarity with a sighting thinning protocol by random sampling (without replacement) of a decreasing fraction of sightings. Better prediction of the distribution of a rarely sighted species occupying a narrow habitat (auk dataset) was expected compared to the distribution of a rarely sighted species occupying a broad habitat (dolphin dataset). We used the original datasets to set up a baseline model and fitted additional models on fewer sightings but keeping effort constant. Model predictive performance was assessed with mean squared error and area under the curve. Predictions provided by the models fitted to the thinned-out datasets were better than a homogeneous spatial distribution down to a threshold of approximately 30 sightings for a GAM with a Tweedie distribution and approximately 130 sightings for the other models. Thinning the sighting data for the taxon with narrower habitats seemed to be less detrimental to model predictive performance than for the broader habitat taxon. To generate reliable habitat modelling predictions for rarely sighted marine predators, our results suggest (1) using GAMs with a Tweedie distribution with presence-absence data and (2) implementing, as a conservative empirical measure, at least 50 sightings in the models.

## Introduction

The rarity of a species can be described in many different ways depending on a combination of criteria such as the extent of its geographic range, the specificity of its habitat and its local abundance (Table 1; [1,2]). According to these criteria, only species that are widely distributed, live in diversified habitats and are abundant, are considered common. Other species are defined as rare because they show a restricted range, a specific habitat, low abundance, or any combination of these criteria.

**Table 1:** The three characteristics that defined the rarity of a species: The habitat specificity, the local abundance and the geographic range (from [1,2]). Each cell defines a form of species rarity except for the top left cell, which characterises a common species.

		**Habitat specificity**			
		Non-specialist		Specialist	
**Local abundance**	High	Common species	Abundant but localised population in several habitats	Abundant and widespread population in specific habitats	Abundant and localised population in specific habitats
	Low	Scarce and widespread population in several habitats	Scarce and localised population in several habitats	Scarce and widespread population in specific habitats	Scarce and localised population in specific habitats
		Large	Limited	Large	Limited
		**Geographic range**			

Many species are naturally rare, but others become rare as a result of man induced pressures; in any case a species rarity contributes to its vulnerability. Therefore, rare species often benefit of a variety of management, conservation or recovery plans to maintain or restore their populations and habitats [3]. Determining the abundance, distribution and habitat use of these species are generally key elements of these plans [4], yet gathering enough high quality data (*e*.*g*., sighting and effort data) is often a challenge.

Rare species usually result in a low number of sightings per unit effort [2]. This scarcity of sighting data renders difficult to fit species distribution models (SDM) because the reliability of predictions largely depends on the number of sightings on which the models are fitted [2,5,6]. Although some studies have addressed the use of models for rare species datasets [2,5,7], the reliability of the predictions produced by these models, and the uncertainty associated with these predictions remain pending issues. To address these issues, one option is to examine how the performance of a species distribution model changes when sighting data become scarcer.

The aim of the present study was to suggest an empirical rule-of-thumb for the minimum sighting number needed to provide reliable predictions for different types of SDMs. This number is expected to be lower for specialist species using a narrow habitat than for more generalist species. It may also vary with the type of residual distribution functions (that is, the likelihood) used when fitting SDM. We thus conducted a sighting thinning experiment using two large datasets (with respect to effort) of marine megafauna collected in the eastern North Atlantic Ocean: small delphinid and auk datasets. Small delphinids are a generalist taxon, and are present at depths between 50–5000 m. In contrast, auks represent a more specialised taxon, as they are present at depths between 10–150 m. Hence, thinning the number of sightings of small delphinids would generate datasets of a rare non-specialist species living in a large geographic range, and the thinning of the number of auk sightings would simulate a rare more specialised species living in a more restricted geographic range. These datasets represent two forms of rare species defined by Rabinowitz ([1]; Table 1). By thinning real datasets, this approach aimed to help habitat modellers circumvent the difficulty associated with assessing the predictive capacity of models fitted to rare species datasets.

## Materials and methods

### Datasets

#### Data collection

**Fig 1:**
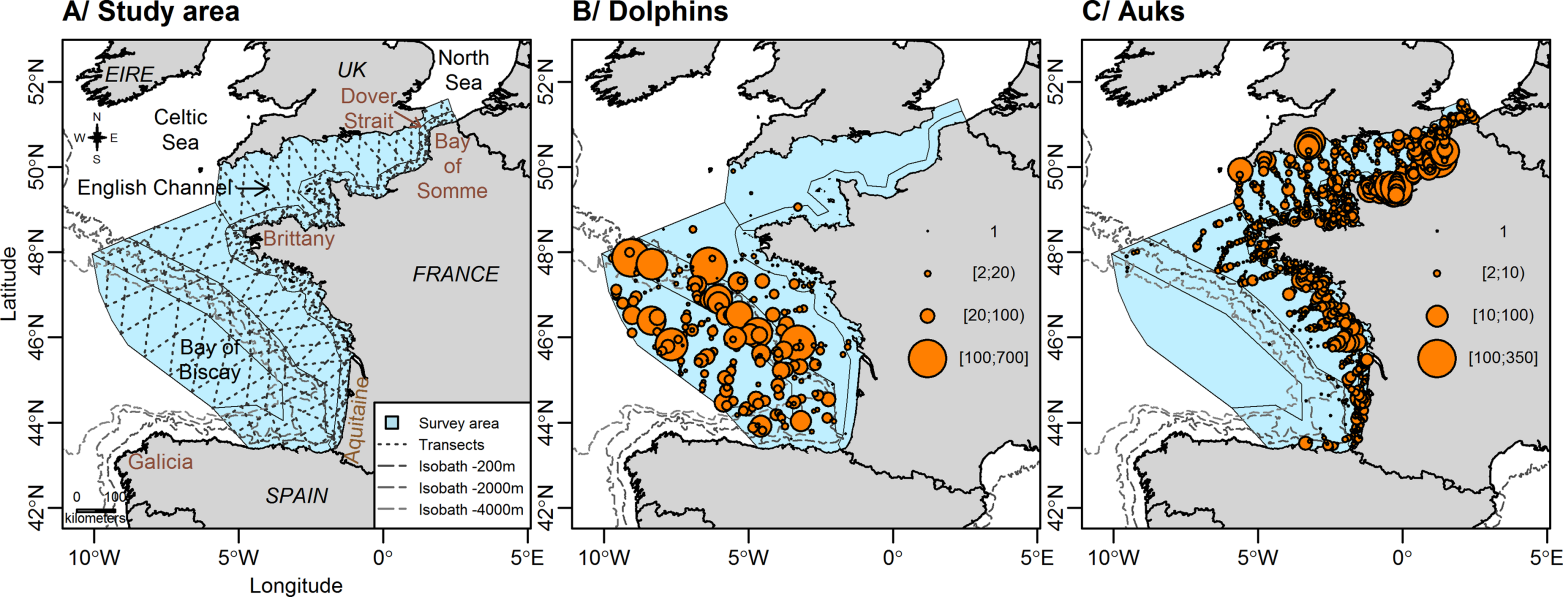
**Study area (A) with dolphin (B) and auk (C) sightings recorded during the survey. The study area expands through the Bay of Biscay and the English Channel.** The surveys were carried out along transects (dotted lines) following a zig-zag pattern across bathymetric strata. The sightings are classified by group sizes, with each point representing one group of individuals.

Marine megafauna sightings were recorded during the two aerial SAMM surveys (*Suivi Aérien de la Mégafaune Marine–*Aerial Census for Marine Megafauna) conducted in the English Channel and the Bay of Biscay (Fig 1). Surveys were conducted during the winter of 2011–2012 (from mid-November to early February; 28,068 km of transects) and the summer of 2012 (from mid-May to early August; 31,427 km of transects). Transects were sampled at a ground speed of 167 km.h^-1^ and an altitude of 182 m. Survey platforms were high-wing aircraft, equipped with bubble windows, allowing observation right under the plane. Sightings and group size were recorded, alongside observation conditions (Beaufort sea-state, glare severity, turbidity, and cloud cover). Transects were subdivided into 10 km-long segments of homogeneous conditions.

In the study, the aim was to use datasets containing enough sightings to implement a data-thinning approach in a realistic and meaningful way. To perform the analysis, we used datasets that were previously exploited and described in various publications [8,9,10,11]. Two taxa with abundant sightings (> 250) and contrasted distributions were selected. The first taxon was composed of small delphinids (hereafter called “dolphins”) including the common (*Delphinus delphis)* and striped (*Stenella coeruleoalba)* dolphins, both of which showing overall offshore distributions. The second taxon was composed of auks (hereafter called “auks”) and mostly consisted of the common guillemot (*Uria aalge)* and, to a much lower extent, the razorbill (*Alca torda)* and the Atlantic puffin (*Fratercula arctica)*, all of which showing a more coastal distribution (Fig 1). A total of 277 dolphin sightings accounting for 14,477 individuals and 1,455 auk sightings representing 16,658 individuals were recorded in good observation conditions (sea state <4 and medium to excellent observation conditions, as defined in [8]) and used in the analyses.

A line-transect methodology was used to record all cetacean sightings [12], while seabird sightings were recorded using a strip-transect methodology [13]. In the line-transect methodology, the angle between the sighting and the track line was measured to determine the effective strip width (ESW; see the detection functions and estimated ESW in [8]) on each side of the plane. In the strip-transect methodology, the sightings were gathered from a 200-m strip on either side of the plane, and it was assumed that all animals within the strip were detected.

#### Environmental predictors

Two categories of environmental predictors at a 10 km resolution were used to model the habitats of the two taxa (Table 2). Static (or physiographic) predictors relate to the bathymetry and included depth and slope, whereas dynamic (or oceanographic) predictors describe the water masses and included the mean, variance and gradient of sea surface temperature (SST); the mean and standard deviation of sea surface height (SSH), and the maximum intensity of general currents (mostly referring to tidal currents in the study area; S1 Fig). To avoid gaps in remotely sensed oceanographic variables, we used a 7-day resolution. All available data were averaged over the 6 days prior to each sampled day (details in [10, 14]).

**Table 2:** Environmental predictors used for habitat modelling.

Environmental predictors	Sources	Effects on pelagic ecosystems of potential interest to top predators
**Physiographic**		
Depth (m)	A	Shallow waters could be associated with high primary production
Slope (°)	A	Associated with currents, high slope induce prey aggregation and/or primary production increasing
**Oceanographic**		
Mean of SST (°C)	B	Variability over time and horizontal gradients of SST reveal front locations, potentially associated to prey aggregations
Variance of SST (°C)	B	
Mean gradient of SST (°C)	B	
Mean of SSH (m)	C	High SSH is associated with high mesoscale activity and prey aggregation and/or primary production increase
Standard deviation of SSH (m)	C	
Daily maximum intensity of the currents (m.s^-1^)	D	High currents induce water mixing and prey aggregation

A: Depth and slope were computed from the GEBCO-08 30 arc-second database (http://www.gebco.net/). B: Mean, variance and gradient of sea surface temperature (SST) were calculated from the ODYSSEA products from Copernicus Marine Environment Monitoring Service (http://marine.copernicus.eu/). C: The MARS 3D model from Previmer ([15]; www.previmer.org) was used to compute mean and standard deviation of sea surface height (SSH). D: Daily maximum current intensity was computed from the MARS 2D model ([15]; www.previmer.org).

### Statistical analyses

#### Analytical strategy

**Fig 2:**
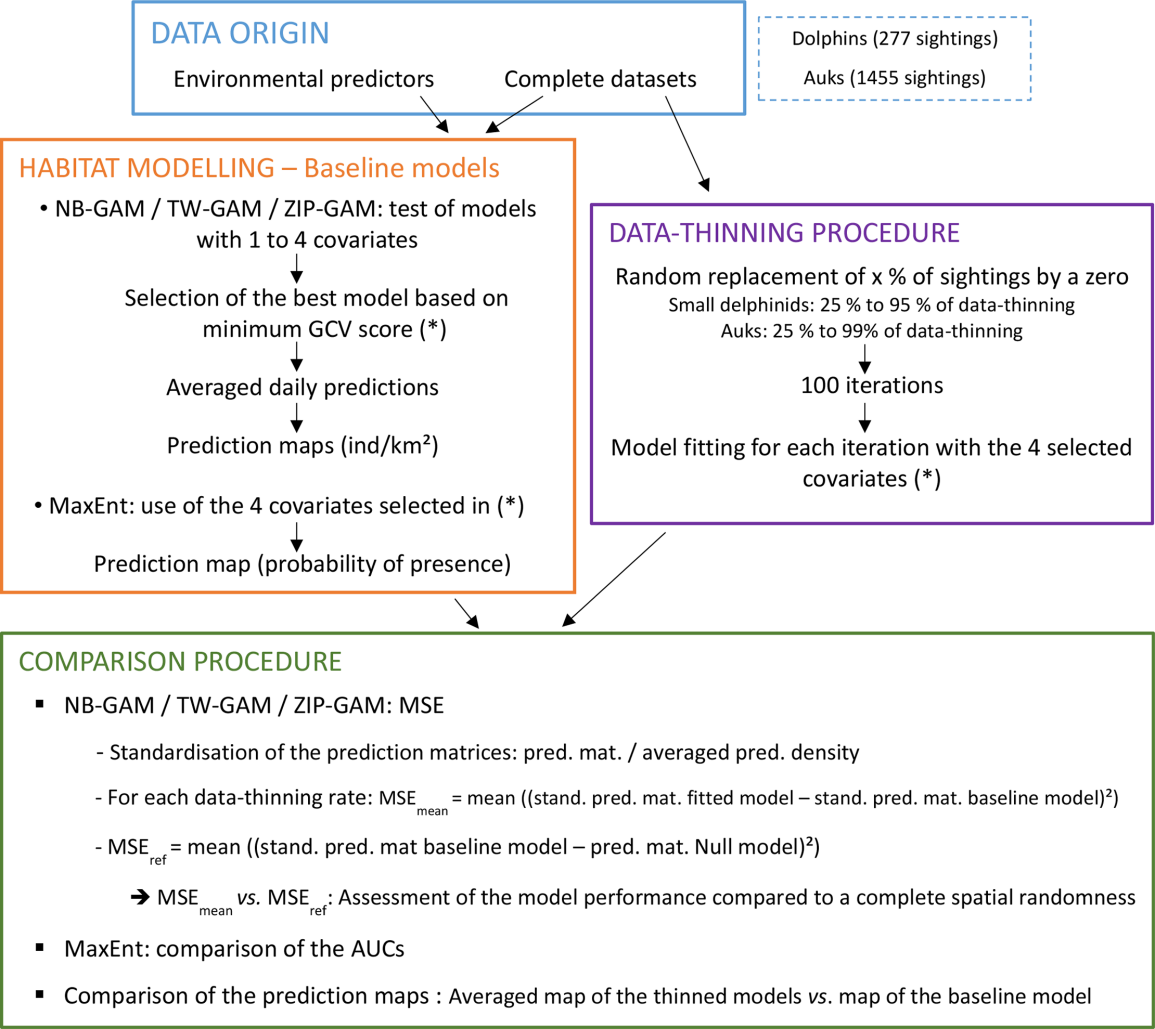
Flowchart of the methods used in the study. NB-GAM: generalised additive model with a negative binomial distribution; TW-GAM: generalised additive model with a Tweedie distribution; ZIP-GAM: generalised additive model with a zero-inflated Poisson distribution; MaxEnt: maximum entropy model; GCV: generalised cross-validation; ind: individuals; MSE: mean squared error; stand.: standardised; pred.: prediction; mat.: matrix; ref: reference; AUC: area under the curve.

We tested the predictive capacity of various SDMs fitted to datasets on rarely sighted species (Fig 2). Two categories of SDMs can be used to predict a species distribution and model its habitats: presence-absence and presence-only models [16]. By establishing the functional relationships between sightings and environmental conditions, presence-absence models (*e*.*g*., Generalised Linear Models, GLMs, or Generalised Additive Models, GAMs) can predict areas of high species occurrence [16–18]. In contrast, with presence-only models (*e*.*g*., Ecological Niche Factor Analysis, ENFA, or Maximum Entropy Modelling, MaxEnt), only sites with environmental conditions similar to those of the sites where the taxon was recorded can be identified [19,20]. Presence-only models are the default option when data on absence (effort data) are not available [21], but the accuracy of presence-only model outputs largely relies on the representativeness of the sampled habitats [22]. Because of its ease-of-use, MaxEnt model is widely used by managers and environmental agencies to help prioritising conservation areas [23–25]. Consequently, assessing the predictive performance of these presence-only models, compared to that of the presence-absence models, is relevant for rare species for which few sightings are typically available unless a considerable amount of effort is deployed.

Three presence-absence models and one presence-only model were tested. We used a GAM with a negative binomial distribution (NB-GAM), a GAM with a Tweedie distribution (TW-GAM), a GAM with a zero-inflated Poisson distribution (ZIP-GAM), and a MaxEnt model. These SDMs were first fitted to the original dolphin and auk datasets in order to select the four most important predictors for each taxon (hereafter referred to as ‘baseline models’). These baseline models served as a reference to compare models fitted to the thinned-out datasets (hereafter referred to as the ‘experimental models’). The original datasets were thinned of sightings by randomly removing 25–99% of the sightings. For each thinning-out level, the four SDMs were fitted with the same explanatory variables as in the baseline model. Finally, predictions from experimental models were compared to those of the baseline models to determine the minimum number of sightings to reliably predict rare species distribution. Although model performance is largely determined by its selected variables [26], we used the same specification for each experimental model in this study to assess how the results were affected by the sighting thinning alone. This choice reflects current practice in marine spatial planning is which the same SDM specification is frequently used by end-users (*e*.*g*. managers) but updated at a much lower frequency by researchers.

#### Baseline models

To fit GAMs, we used the ‘gam,’ ‘nb,’ ‘tw’ and ‘ziP’ functions within the ‘mgcv’ package [27,28] (See S1 Additional for more details about the models). A log function linked the response variable to the additive predictors; the curve smoothing functions were restricted to three degrees of freedom [29]; finally an offset that considered the variation of effort per segment [30] was included and calculated as segment length multiplied by 2*ESW (see Laran et al. [8]). After removing all combinations of variables with correlation coefficients higher than |0.7|, the models with combinations of 1 to 4 variables were tested [31,32], and the best models were selected, *i*.*e*., the models with the lowest generalised cross-validation score (GCV; [33]), which estimates the mean prediction error using a leave-one-out cross-validation process [34]. For both taxa, the selected variables for NB-GAM, TW-GAM and ZIP-GAM were identical so that it was straightforward to compare the different models.

For each fitted model, predicted densities (in individuals per km^2^) were mapped on a 0.05°x0.05° resolution grid. We computed the predictions for each day of the surveys and averaged the predictions over the entire survey period. To limit extrapolation, the covariates were constrained within the range of the covariate values used when fitting the models. Finally, we provided uncertainty maps by computing the variance around the predictions as the sum of the variance around the mean prediction and the mean of the daily variances. Then, the coefficient of variation was calculated as

$$CV=100\times \sqrt{(variance}\;over\;the\;survey\;period)/mean\;over\;the\;survey\;period.$$

We also tested the effect of thinning-out on a presence-only model: Maxent (version 3.3.3, http://www.cs.princeton.edu/~schapire/maxent/; [24]). In this model, environmental relationships are estimated using the background samples of the environment instead of absence locations [35]. For both taxa, we first removed all absences from the input files used for the presence-absence models to obtain a file with only presence locations that would be compatible with the software. We used the four environmental variables determined by the selection procedure for the GAMs to allow for comparisons between the different models. Finally, we selected a “*hinge*” feature as a model parameter to generate models with smooth functions similar to GAMs with a default prevalence of 0.5 and a logistic output format to obtain a probability of presence of the species groups [35–37]. Table 3 summarises the tested models and their characteristics.

**Table 3:** Details of the models used in the study.

Models	Used names	Data	Settings and details
**Generalised Additive Model with Negative Binomial distribution**	NB-GAM	Over-dispersed PA	Used R-3.1.2*, package **mgcv**, function GAM, *Negative Binomial* distribution, log-link function, included an offset, 3 degrees of freedom for the smoothing curve functions
**Generalised Additive Model with Tweedie distribution**	TW-GAM	Over-dispersed PA	Used R-3.1.2*, package **mgcv**, function GAM, *Tweedie* distribution, log-link function, included an offset, 3 degrees of freedom for the smoothing curve functions
**Generalised Additive Model with Zero-Inflated Poisson distribution**	ZIP-GAM	Zero-inflated PA	Used R-3.1.2*, package **mgcv**, function GAM, *ZIP* distribution, log-link function, included an offset, 3 degrees of freedom for the smoothing curve functions
**Maximum Entropy Modelling**	MaxEnt	Presence-only	Used MaxEnt software version 3.3.3, *hinge* feature, default prevalence of 0.5, logistic output format

GAM: generalised additive model; GLM: generalised linear model; PO: Poisson; NB: negative binomial; TW: Tweedie; ZIP: zero-inflated Poisson; PA: presence-absence data; AIC: Akaike information criterion.

* R Core Team [38]

#### Thinning-out of the sightings

To generate datasets of rare species, we thinned the original auk and dolphin sightings at different rates to simulate rare species datasets with about ten sightings. We aimed to obtain a decreasing number of sightings, simulating thereby an increasing rarity of the two taxa under a constant sampling effort. In the dolphin dataset, we randomly replaced 25, 50, 75, 90, 92 and 95% of the sightings with zeros, and in the auk dataset, we randomly replaced 25, 50, 75, 90, 92, 95, 97 and 99% of the sightings with zeros (Table 4 and S2 and S3 Figs show examples of thinning-out). For each thinning rate, sightings to be replaced with zero were randomly sampled without replacement. To ensure a homogeneous sighting thinning in the sampling area, 100 datasets were randomly created from the complete datasets for each degradation rate, hence producing 100 randomly thinned or experimental datasets for each thinning rate. This procedure simulates different levels of species rarity as observed under a constant sampling effort. Removing part of survey effort (*e*.*g*., whole transects) would not have generated a greater rarity of the species but only a lower sighting effort; and would have led to similar results of the baseline models because encounter rates had remained similar on average.

**Table 4:** Number of sightings contained in the thinned or experimental datasets for each thinning rate and each species group.

		Thinning rates								
Species groups		Original	25%	50%	75%	90%	92%	95%	97%	99%
**Dolphins**	n_sigh_	277	208	139	69	28	23	14	-	-
	n_z_	3043	3112	3181	3250	3292	3297	3306	-	-
	%_z_	91.7	93.7	95.8	97.9	99.2	99.3	99.6	-	-
**Auks**	n_sigh_	1455	1091	728	364	146	116	73	44	15
	n_z_	2046	2409	2773	3137	3355	3384	3428	3457	3486
	%_z_	56	66	76	85.9	91.9	92.7	94	94.7	95.5

n_sigh_: number of sightings; n_z_: number of segments with a zero; %_z_: percentage of zeros; Original: initial (and complete) datasets. “–”indicates that the sighting thinning was not performed.

#### Assessment of the predictive performance of the model

The baseline SDMs were selected using the minimum GCV score, and a leave-one-out cross-validation process was used to estimate mean prediction error and explained deviances [31,32]. However, for experimental models, we based the assessment of the predictive performance of the presence-absence models on two criteria: mean squared error (MSE; [37,38]) and maps of the predicted densities. The MSE directly compared the prediction matrices of the experimental models to the prediction matrix of the baseline model. Each cell of the matrices provides the densities predicted by the model over the entire prediction area. The MSE is given by $MSE=mean\;\left(\sum {\left({\hat{Y}}_{exp}-{\hat{Y}}_{baseline}\right)}^{2}\right)$ [39,40]. Here, “${\hat{Y}}_{exp}$*”* represents the prediction matrix of an experimental model, and “${\hat{Y}}_{baseline}$*”* represents the prediction matrix of the baseline model. For each type of model and thinning rate, we averaged the MSEs of all the experimental models to obtain an averaged MSE (called MSE_mean_). Then, we investigated whether the predictions provided by the models fitted to sighting thinned-out datasets were better than those from a homogeneous process. For this purpose, we compared the MSE of each fitted model and the MSE_mean_ to a reference threshold, called the MSE_ref_, which was calculated as the MSE between the prediction matrix of the baseline model (NB-GAM, TW-GAM or ZIP-GAM) and the prediction matrix of a null NB-GAM, TW-GAM or ZIP-GAM (which described a homogeneous spatial distribution). We assumed that if the MSE was higher than the MSE_ref_, it was more appropriate to consider a homogeneous spatial distribution rather than taking into account the predictions provided by the experimental model.

To assess the predictive performance of the MaxEnt models, we used the area under the receiver operating characteristic curve (AUC; [19]). AUC allows for the direct comparison of SDM predictive performance but can only be used on binary data. An AUC of 1 indicates a perfect discrimination between the sites where the species is present and absent, an AUC of 0.5 indicates a discrimination equivalent to a random distribution, and an AUC lower than 0.5 indicates that the model performance is worse than a random guess [19]. We compared the AUC of each fitted model and the AUC_mean_ (averaged over the 100 fitted models) to the AUC of the baseline model and used a threshold value of 0.5 to assess the performance of the experimental models.

Finally, we compared the prediction maps of the models fitted to the thinned datasets to the prediction maps of the baseline models in order to determine the lowest sample size that did not change predicted distribution patterns. For each model type and each thinning rate, we averaged the predictions over the 100 models fitted to the thinned datasets and produced averaged prediction maps that we compared to the prediction maps of the baseline models. We averaged the predictions over the 100 fitted models to ensure a uniform data deletion throughout the area. In practice, habitat modellers only have one real dataset (not 100); hence, we compared the MSE or AUC of each fitted model to the MSE_ref_ or AUC_ref_ to determine the proportion of the model that provided good predictions.

## Results

### Model selection and predictions of the baseline models

#### Small delphinids

**Fig 3:**
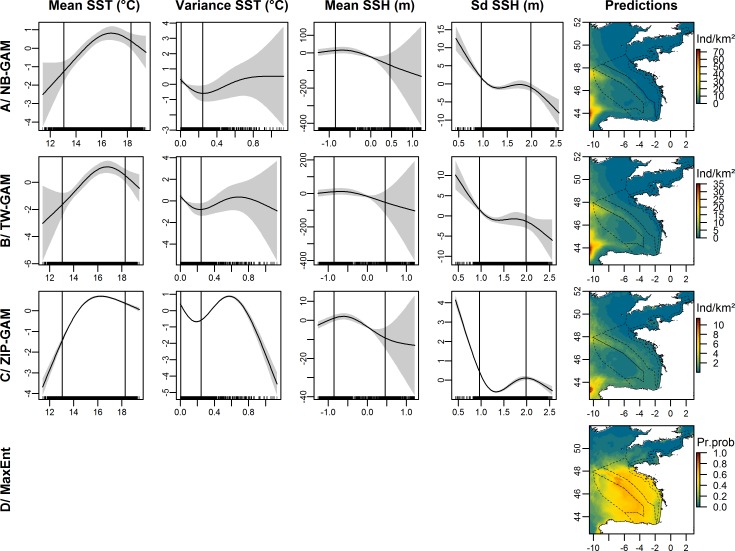
Estimated smooth functions for the selected covariates and predicted distribution of dolphins in individuals.km^-2^ (Ind/km^2^) for each presence-absence model and in presence probabilities (Pr.prob) for the Maxent model. The solid line in each plot is the estimated smooth function, and the shaded regions represent the approximate 95% confidence intervals. The y-axis indicates the number of individuals on a log scale, and a zero indicates no effect of the covariate. The best model fits are between the vertical lines indicating the 10^th^ and 90^th^ quantiles of the data. The dotted lines represent the bathymetric strata of the survey area. The white areas on certain maps represent the absence of predictions beyond the range of covariates used in fitted models.

The explained deviances in the dolphin dataset were moderate to fairly high: 38.4% for the NB-GAM, 37.3% for the TW-GAM and 17.1% for the ZIP-GAM. Densities were best predicted from SST mean and variance and SSH mean and standard deviation (Fig 3). All models showed similarly-shaped smooth functions, with the highest densities of delphinids predicted at temperatures of approximately 16°C (variance close to 0°C) and low average altimetry (SSH, approximately -0.5 m, standard error approximately 0.5 m). Small delphinids were predicted to be distributed in offshore waters from the continental shelf to oceanic waters with higher densities along the slope and a peak north of Galicia (Fig 3). There was a strong match between sightings and model predictions (Figs 1 and 3) with high predicted densities associated with low coefficients of variation (S4 Fig). With an AUC of 0.822, the MaxEnt model correctly predicted the presence probabilities of delphinids. Similar to the other fitted models, the highest presence probabilities were predicted along the slope of the Bay of Biscay and were evenly distributed elsewhere (Fig 3).

#### Auks

**Fig 4:**
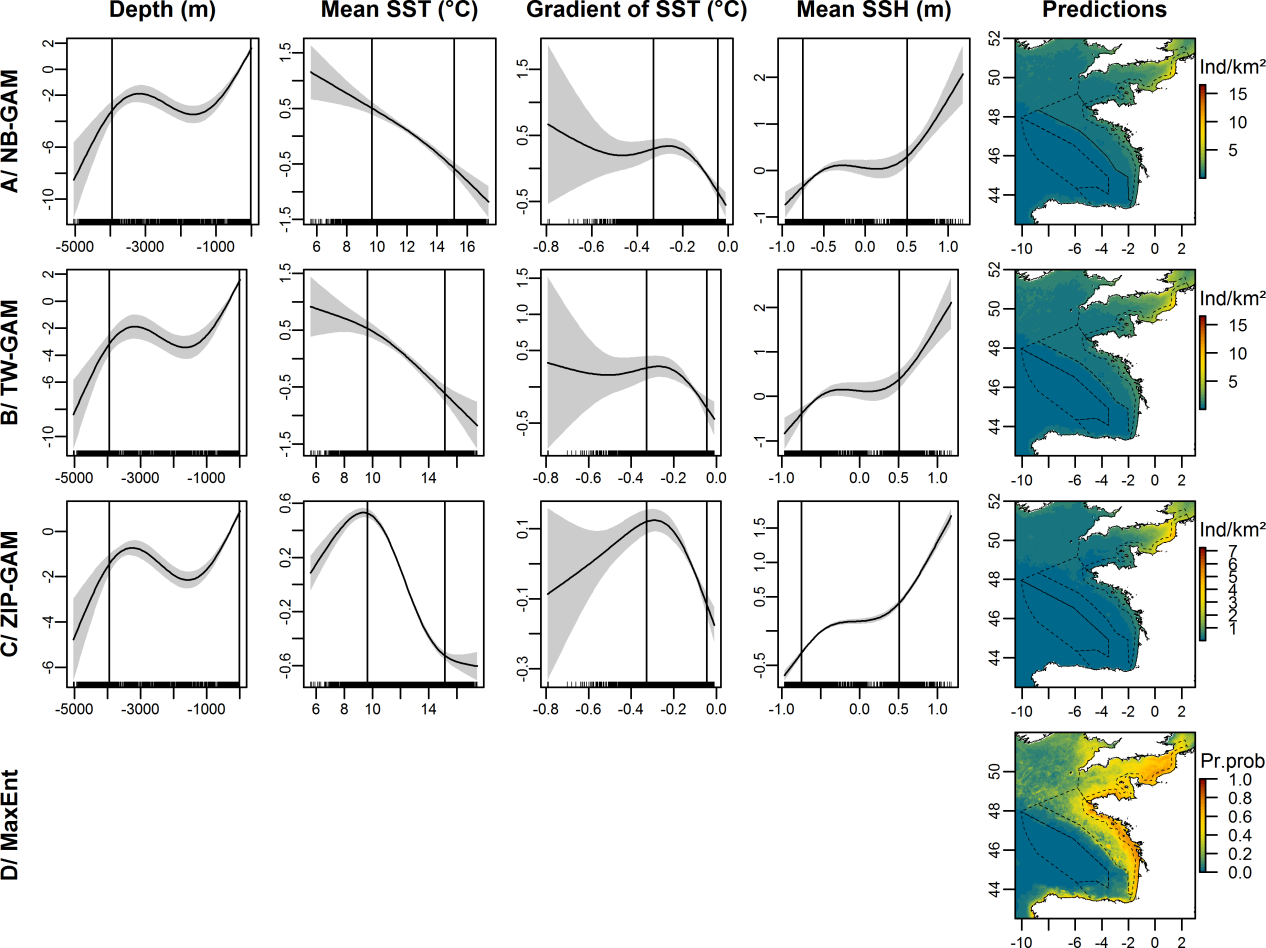
Estimated smooth functions for the selected covariates and predicted distribution of auks in individuals.km^-2^ (Ind/km^2^) for each presence-absence model and in presence probabilities (Pr.prob) for the Maxent model. The solid line in each plot is the estimated smooth function, and the shaded regions represent the approximate 95% confidence intervals. The y-axis indicates the number of individuals on a log scale, and a zero indicates no effect of the covariate. The best model fits are between the vertical lines indicating the 10^th^ and 90^th^ quantiles of the data. The dotted lines represent the bathymetric strata of the survey area.

The explained deviances in the auk dataset reached 44.9% for the NB-GAM, 40.9% for the TW-GAM and 33.6% for the ZIP-GAM. The variables selected by the three baseline models were depth, mean and gradient of SST and mean SSH (Fig 4). Greater auk densities were associated with colder and shallower waters, stronger gradients of temperature and higher positive altimetry. The predicted distribution ranged from the coast to the edge of the continental shelf and predicted densities were particularly high in the eastern English Channel (Fig 4). There was a good match between the sightings and the predictions of the model (Figs 1 and 4) with high predicted densities associated with low coefficients of variation (S4 Fig). The MaxEnt model, with an AUC of 0.842, generally predicted the same distribution as the other models with higher concentrations along the coast (Fig 4).

### Predictive performance of the experimental models

#### Small delphinids

**Fig 5:**
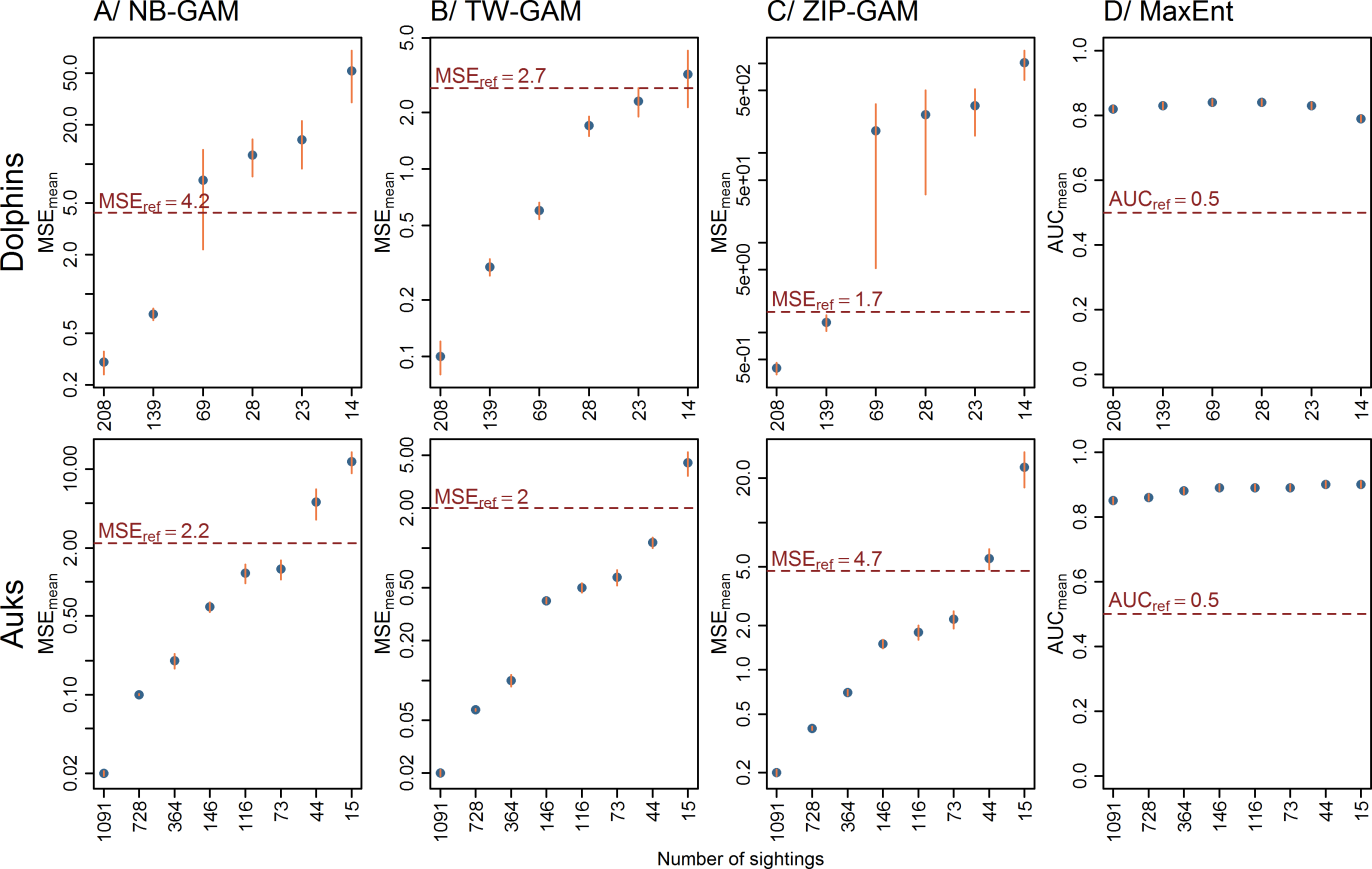
Evaluation of the predictive performance of the models using MSE and AUC. MSE_mean_: mean squared error averaged over 100 models; AUC_mean_: area under the curve averaged over 100 models; Ref: reference index (*i*.*e*., a homogeneous spatial distribution). A log scale is applied on the y-axis. The vertical bars on each point represent the standard error calculated from 100 models.

**Fig 6:**
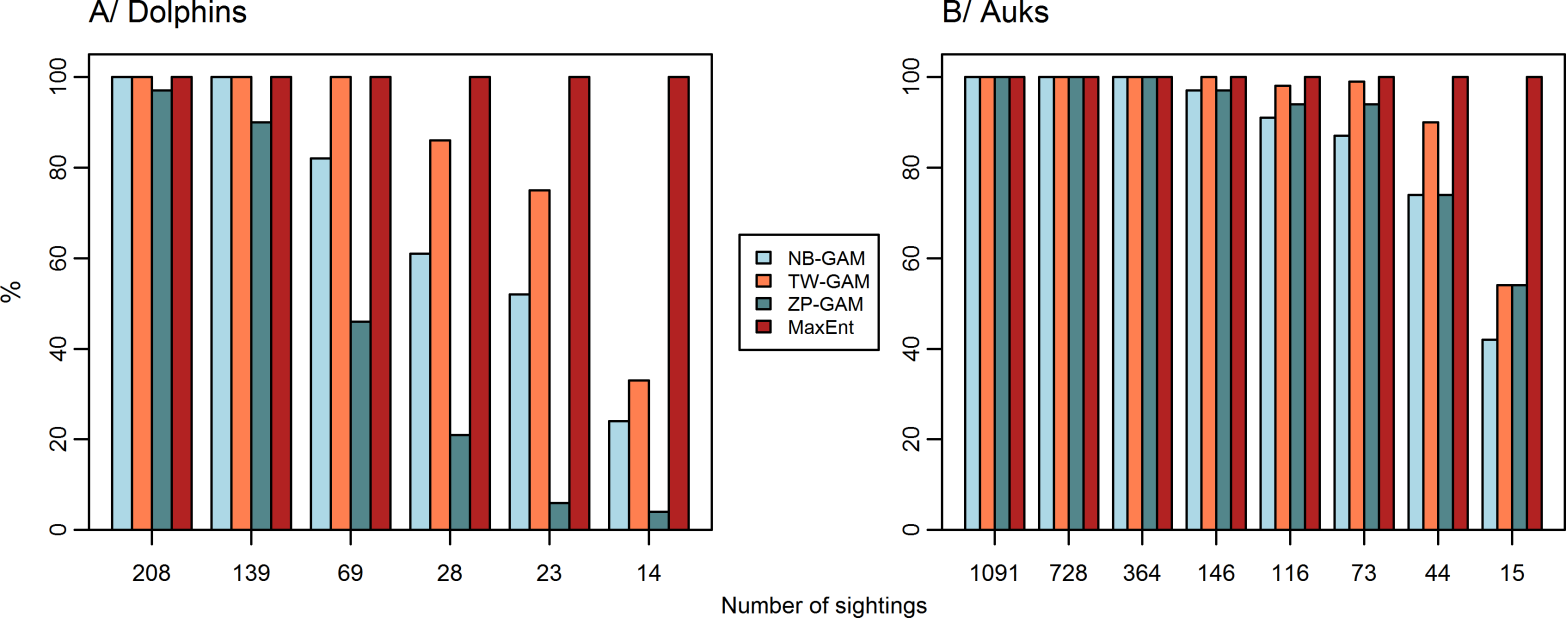
Proportion of experimental models better than a homogeneous spatial distribution. Each bar represents, the proportion of the experimental models out of the 100 fitted in which the MSE is lower than the MSE_ref_ for each number of sightings, *i*.*e*., the model that is better than a homogeneous spatial distribution. Each colour represents a different model type.

As expected, a decrease in the number of sightings led to an increase in MSE_mean_ (Fig 5). Predictions with 208 sightings (the lowest thinning rate) were closer to those of the baseline models than the predictions with only 14 sightings (the highest thinning rate). The comparison of MSE_mean_ with MSE_ref_ (representing the MSE between the baseline predictions and the null models), suggested that for less than 139 sightings, MSE_mean_ values for NB-GAMs and ZIP-GAMs were higher than MSE_ref_. In contrast, MSE_mean_ values for TW-GAMs were lower than MSE_ref_, except for the most extreme thinning rates that yielded as few as 14 sightings. Consequently, below 139 sightings, it was better to predict a homogeneous spatial distribution rather than to use the predictions provided by the NB-GAMs and the ZIP-GAMs. For the TW-GAMs, this threshold was under 23 sightings. Furthermore, the number of experimental models in which the MSE was higher than the MSE_ref_ varied among model types (Fig 6). With a decrease in the number of sightings, the proportion of experimental models in which predictions were better than a homogeneous spatial distribution decreased (MSE<MSE_ref_; Fig 6). For example, with 23 sightings, only 51% NB-GAMs and 6% ZIP-GAMs predicted better than a homogeneous spatial distribution compared to 75% TW-GAMs. For MaxEnt, AUC_mean_ values of the experimental models were high (>0.82) and very similar and higher than the AUC_ref_, which predicted a homogeneous distribution of the sites occupied by the species.

**Fig 7:**
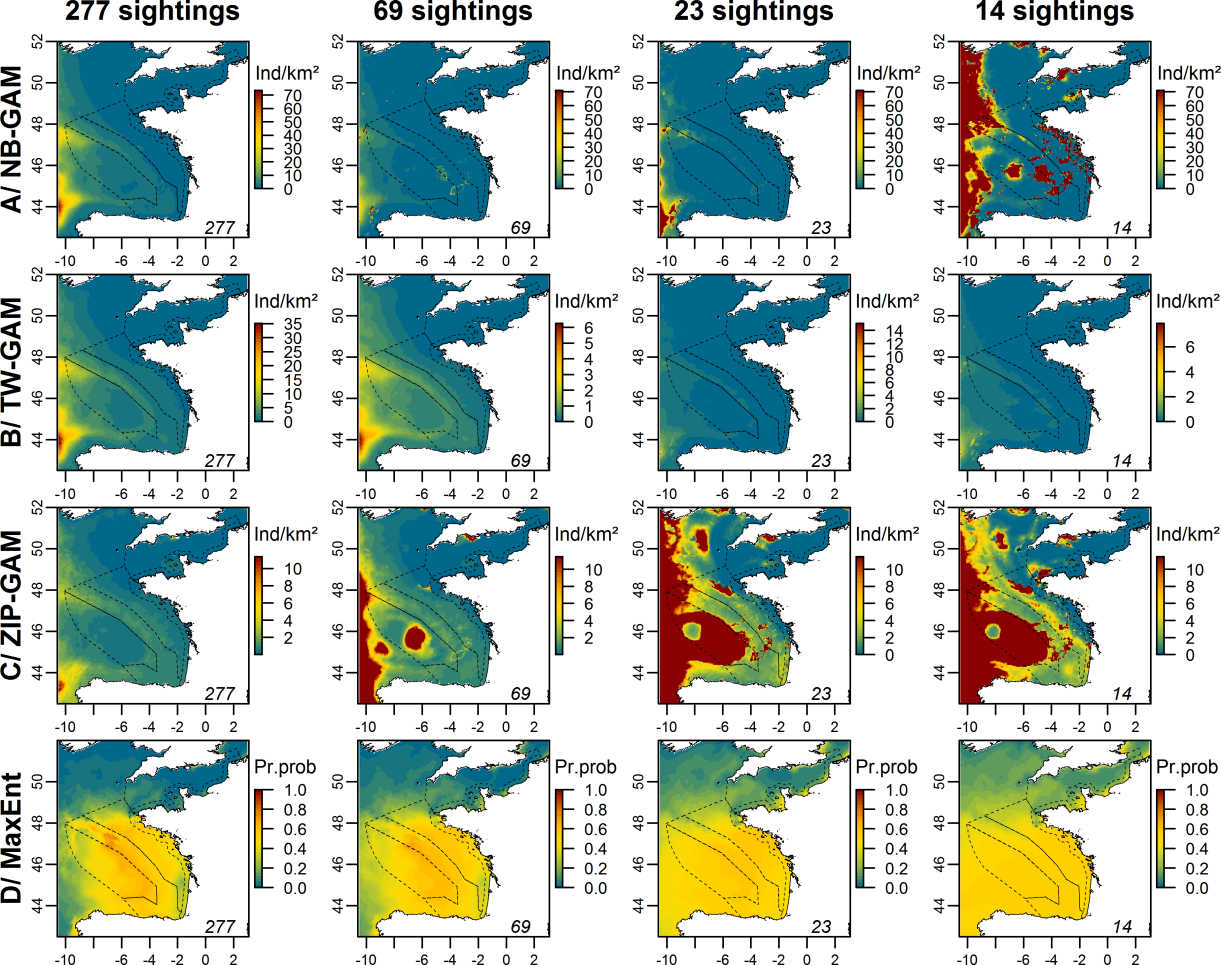
Prediction maps of dolphins averaged over 100 models fitted to thinned datasets for each type of model in the Bay of Biscay and the English Channel. The rows represent the different types of generic models, and the columns represent the number of sightings used to fit the models. The numbers in the right corner of each map represent the number of sightings used to fit the model. The scale is in individuals.km^-2^ (Ind/km^2^) for the NB-GAM, the TW-GAM and the ZIP-GAM and in the probability of presence (Pr.prob) for MaxEnt. This figure only shows the results for which a change was observed compared with the other predictions. All maps are presented in S5 and S6 Figs. The dotted lines represent the bathymetric strata of the survey area.

We noticed an important variation in the prediction maps among experimental models (Fig 7; S5 and S6 Figs). Despite a decrease in the number of sightings, the distribution patterns of the baseline models were maintained down to 139 sightings for NB-GAMs and ZIP-GAMs. Beyond this threshold, the pattern disappeared or became unrealistic. Predictions from TW-GAM were similar to the distribution pattern of the baseline model with as few as 28 sightings. Beyond this threshold, the pattern started to fade out. When compared to the baseline, the highest densities predicted by NB-GAM, TW-GAM and ZIP-GAM were associated with the highest uncertainties (S7 and S8 Figs).

Relative presence probability predicted by MaxEnt model became more uniform in area when the number of sightings decreased, with high probability areas located along the slope, and low probability areas located near the Aquitaine coast gradually fading out (Fig 7).

#### Auks

MSE_mean_ values increased with decreasing numbers of sightings (Fig 5). As expected, predictions with 1,091 sightings (the lowest thinning level) were closer to those of the baseline model (1,455 sightings) than were the predictions with only 15 sightings (the highest thinning level). When the number of sightings was lower than 73, MSE_mean_ values of NB-GAMs and ZIP-GAMs were higher than MSE_ref_, whereas MSE_mean_ for TW-GAMs was higher than MSE_ref_ with only 15 sightings. Consequently, with less than 73 sightings, the predictions provided by NB-GAMs and ZIP-GAMs were worse than a homogeneous spatial distribution. For TW-GAMs, this threshold was below 44 sightings. Similar to the results for dolphins, the number of models in which the MSE was higher than the MSE_ref_ varied (Fig 6). With 15 sightings, only 42% NB-GAMs compared to 54% TW-GAMs and ZIP-GAMs predicted better than a homogeneous spatial distribution. The AUC_mean_ values for the MaxEnt model were very high (>0.85) and slightly increased with a decreasing number of sightings. Overall, the AUC_mean_ values were higher than AUC_ref_ (Fig 5).

**Fig 8:**
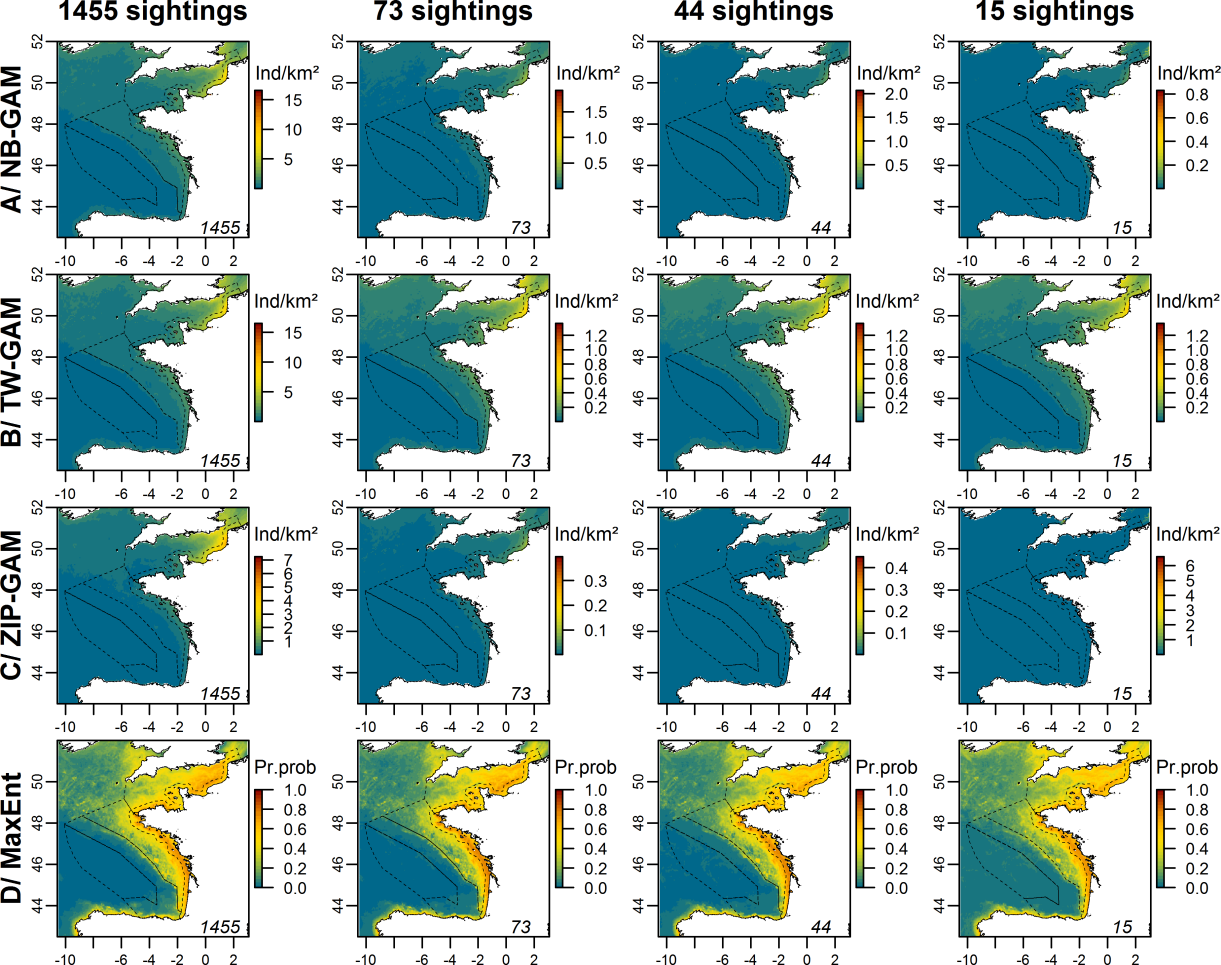
Prediction maps of auks averaged over the 100 models fitted to thinned datasets for each type of model in the Bay of Biscay and the English Channel. The rows represent the different types of generic models, and the columns represent the number of sightings used to fit the models. The numbers in the right corner of each map represent the number of sightings used to fit the model. The scale is in individuals.km^-2^ (Ind/km^2^) for the NB-GAM, the TW-GAM and the ZIP-GAM and in the probability of presence (Pr.prob) for the MaxEnt model. This figure only shows the results for which a change was observed compared with the other predictions. All maps are presented in S9 and S10 Figs. The dotted lines represent the bathymetric strata of the survey area.

We noticed clear distinctions in averaged prediction maps between experimental models (Fig 8; S9 and S10 Figs). For NB-GAMs, the prediction patterns were maintained down to 116 sightings, but under this threshold, patterns gradually disappeared. Despite a decrease in predicted densities, the distribution patterns predicted by the TW-GAMs remained the same down to 15 sightings. The distribution patterns predicted by ZIP-GAMs progressively disappeared below 364 sightings. Higher densities predicted by NB-GAMs, TW-GAMs and ZIP-GAMs fitted to thinned datasets were associated with lower uncertainties (S11 and S12 Figs). Furthermore, uncertainties of TW-GAMs were lower than those of NB-GAMs and ZIP-GAMs. The MaxEnt models showed some homogenisation of the distribution patterns with a decreasing number of sightings, but the general pattern was maintained no matter the number of sightings (Fig 8).

## Discussion

### General considerations

To determine the model that would best predict the distribution of a rare species, we compared different types of models, both presence-absence and presence-only models. We assessed the predictive performance of a known model using a reduced amount of available sighting data. Our findings suggest that the habitats for species that are rare or seldom seen are best described using a GAM with a Tweedie distribution (if effort data are available). GAMs with a negative binomial or zero-inflated Poisson distribution and MaxEnt models became inadequate for dataset under 130 sightings while TW-GAMs kept performing well down to a sample size of 30 sightings.

#### Biological systems

Dolphins, including common and striped dolphins, and auks, including common guillemot, razorbill and Atlantic puffin, were used as biological models for two reasons. First, sightings of these taxa were large enough to allow both standard statistical analyses and thinning to be conducted (277 dolphin sightings and 1,455 auk sightings). Second, dolphins and auks in the Bay of Biscay show well-defined and distinct patterns of distribution [10], which allows evaluating the predictive accuracy of the models.

Species groups pooled different species because of the difficulty to distinguish individuals at a species level from air. Pooling species into groups probably create categories with a broader habitat than the habitat of any of the constituting species, resulting in slightly larger sample size recommendations. However, the auk taxon is mainly dominated by the common guillemot and distribution patterns obtained in the study would mainly represent the common guillemot winter distribution. Indeed, auks wintering in the Bay of Biscay mostly originate from colonies located in the British Isles, where breeding populations of razorbill amount to 187,000 individuals, Atlantic puffin to 580,000 individuals and common guillemot to 1,416,000 individuals [41]. Concerning dolphins, combining the two species resulted in a bimodal habitat. Indeed, shipboard surveys (CODA; partly SCANS-II and SCANS-III) revealed how the two species are present in all offshore habitats, yet the common dolphin predominates over the shelf and the shelf-break, whereas the striped dolphin is more frequent in oceanic waters. Consequently, this species complex would reflect some kind of bimodal habitat characteristics that can be found *in natura*, as for instance in some delphinids like the bottlenose dolphin (*Tursiops truncatus*) with its pelagic and coastal ecotypes [42].

Auks and dolphins differ widely in their habitat specificity, particularly regarding depth, an environmental variable of major importance to characterise marine habitats. Hence, the sighting thinning experiment conducted in both taxa simulated two different cases of rarity (Table 1). Thinning small delphinid sightings simulate a rare non-specialist species living in a broad habitat (row 2, column 1 of Table 1) while thinning auk sightings generate a rare specialist species living in a narrower habitat (row 2 and column 4 in Table 1). Modelling the habitat of the species described in the first row of Table 1 is not challenged by the number of sightings as the species is locally abundant, but is challenged by the location of the survey (if the survey was outside the core distribution of a species, sighting data would be scarce). Consequently, only habitat modelling for rare species of the second row of Table 1 remain an issue. To provide a more complete answer regarding the sample sizes needed to characterise pelagic animal distributions, further analyses and meta-analyses with multiple and diversified datasets should be conducted to obtain robust recommendations. We are aware that determining the number of data needed to model the rare species habitats is an important challenge, for example to inform field efforts, but that a single study cannot consider all possible cases. An alternative research avenue would be to use virtual species instead of real species [43], which would allow to control all the conditions of the procedure but would not reflect the complex reality of the ecosystems. A methodology can work with a virtual species but fail in a real case.

#### Baseline models

To assess the effect of the number of sightings, we tested three presence-absence models, NB-GAM, TW-GAM and ZIP-GAM, and one presence-only model, the MaxEnt model. All models tested in this study can handle datasets with many zeros but in different ways. We also wanted to test a presence-only model because in the case of rare and elusive species, opportunistic data, which represent a common example of presence-only data, often represent the majority of available data [44]. The MaxEnt model is able to model complex interactions between the response and the predictor variables [19,35,45], has been reported to be appropriate for presence-only datasets [46] and is widely used in species conservation planning due to its simplicity of use [23–25].

Variable selection was only performed on the baseline models. As the performance of a model is largely controlled by its selected variables [26], the models that used thinned-out sightings might be biased and are suboptimal (because some sighting data are ignored). Indeed, variables selected by a model fitted to few data could differ from models fitted to much larger datasets. However, we did not attempt to find the best model fit but to test the robustness of model predictions to thinning; variable selection was, to a certain extent, secondary to our purposes. In an ideal situation, the habitat of the species is known *a priori*. In practice, this is rarely the case, but in realistic situations, a SDM is first developed and then used repeatedly until the need to update it becomes an imperative. Thus the same SDM specification may be used without undergoing rounds of variable selection each time a new datum is added to an existing dataset. In a similar fashion, while MSE give guarantee on the predictive performance on average (*i*.*e*. under repeated use of the same model with different data generated from the same process), more often than not a single dataset is available for a given area. Consequently, to approximate a real situation in which one needs to model rare species habitats from a single dataset, the predictive capacity of each experimental model has been assessed in order to determine the probability for a single experimental model to reproduce the baseline model predictions.

#### Thinning-out sighting data

Thinning rates applied in this study were arbitrarily determined to obtain, in the most extreme scenario, as few as 15–20 sightings, which is a threshold commonly observed for very rare species, particularly in marine megafauna [47,48]. Overfitting can be an issue with small datasets, *i*.*e*., the selected model becomes too complex compared to the number of implemented sightings [49,50]. Particularly, overfitting could have occurred in the models with the highest thinning rates. Nonetheless, the NB-GAM, the TW-GAM and the ZIP-GAM performed differently with the same small number of sightings (14–15 sightings). The NB-GAM and the ZIP-GAM did not manage to predict distribution patterns consistent with the baseline models, whereas the TW-GAM did.

#### Predicting habitats of rare species

Our aims were to assess the robustness of predictions from different SDMs by assessing prediction invariance under increasing levels of thinning of sightings used in model fitting. Overall, predictive robustness differed between SDMs. All distributions predicted by the MaxEnt model were better than a homogeneous spatial distribution. There was, however, a gradual homogenisation of predicted dolphin presence probabilities over the whole area with increasing thinning rates. With very few sightings (approximately 28), MaxEnt was no longer able to distinguish key areas of either high or low presence probabilities. In contrast, despite some homogenisation of the predicted probabilities, auk distribution patterns were correctly predicted, even with as few as 15 sightings. Consequently, thinning affected model predictive performance differently whether the studied taxon was a generalist or specialist one. However, these results not be truly representative of the empirical performance of MaxEnt. Our data were collected with a standard protocol that ensured a balanced coverage over the Bay of Biscay, which conforms to the assumptions underlying the appropriate use of presence-only models [22]. This may not be the general case with presence-only data, where survey effort is often biased. Despite a balanced sampling effort in the field, MaxEnt did not provide satisfactory results for the highest thinning rates, calling into question its use for rare species.

Because thinning sightings emulate false absences (that is a zero observation due for example to imperfect detection in a nevertheless suitable habitat), we expected a better performance by the ZIP-GAM. However, the results were less reliable than those obtained with a TW-GAM. Below approximately 130 sightings, the predicted distributions of the ZIP-GAM were unreliable compared to the predictions of the baseline model, whereas this threshold was as low as approximately 30 sightings for the TW-GAM. This difference is likely due to the current parametrisation of the ZIP family in the ‘mgcv’ package [27,28]. In fact, the current parametrisation uses the linear predictors and linearly scales them on a logit scale to generate extra zero observations (see the help pages in mgcv v1.8–9; [28]). This parametrisation implicitly assumes that the areas with lower densities have a higher probability of non-detection. However, the parameterisation does not allow for incorporating detection-specific covariates, which may better explain the non-detection patterns. Similarly, the NB-GAM provided less convincing results and unreliable predicted distribution patterns compared to the baseline model below approximately 130 sightings.

Even if the TW-GAM provided good results with approximately 20–25 sightings, the results were based on the averages of 100 fitted models and hid substantial variations. In practice, habitat modellers have only one dataset. Therefore, we assessed the individual performance of each experimental model by computing the number of models in which the MSE was higher than the MSE_ref_ and by examining the explained deviances of each experimental model (results not shown). It appeared that with 20 sightings, approximately 50 of the 100 experimental TW-GAMs predicted better than a homogeneous spatial distribution of the two species groups whereas with 40 sightings, 90 of the 100 experimental models provided reliable results (Fig 6). Moreover, by examining the explained deviances for each experimental Tweedie model (results not shown), we found that explained deviances of the experimental models fitted to 28 and 69 sightings for dolphins were good (30–50%). For the smallest number of data (15 and 23 sightings), the explained deviances were very high (>50%) which suggested overfitting. Consequently, to obtain robust predictions, a number of 50 sightings would represent a conservative empirical measure under a homogeneous sampling effort over the study area. However, this number is only valid for the TW-GAM because with the NB-GAM and the ZIP-GAM, the threshold for which all experimental models provided good results (better than a homogeneous spatial distribution) was 100 sightings (Fig 6).

Finally, this study provided a first answer to the question commonly asked by habitat modellers: “What model should be used when studying rare species?" If modellers only have presence data, MaxEnt could be used but with great caution and preferably for specialist species with restricted distributions. With effort data, we would recommend using a GAM with a Tweedie distribution and a minimum of 50 sightings, which is a conservative empirical measure.

## Supporting information

S1 Additional Key concepts about the models used in the study. (PDF)

S1 Fig Maps of averaged covariates over the entire survey. (TIF)

S2 Fig Sighting thinning example for the dolphin dataset. Sightings are classified by group sizes (1; 2–20; 20–100 and 100–700 individuals) with each point representing a group of individuals.
(TIF)

S3 Fig Sighting thinning example for the auk dataset. Sightings are classified by group sizes (1; 2–10; 10–100 and 100–350 individuals) with each point representing a group of individuals.
(TIF)

S4 Fig Uncertainty maps of baseline models. Uncertainty maps representing the coefficient of variation in % associated with the predictive relative density of dolphin and auk groups. Dotted lines represent the survey area.
(TIFF)

S5 Fig Prediction maps of dolphins averaged over 100 models fitted to thinned datasets for each type of model from 25 to 75% of sighting thinning. The rows represent the different types of generic models, and the columns represent the number of sightings used to fit the models. The numbers in the right corner of each map represent the number of sightings used to fit the model. The scale is in individuals.km-2 (Ind/km^2^) for the NB-GAM, the TW-GAM and the ZIP-GAM and in the probability of presence (Pr.prob) for MaxEnt.
(TIFF)

S6 Fig Prediction maps of dolphins averaged over 100 models fitted to thinned datasets for each type of model from 90 to 95% of sighting thinning. The rows represent the different types of generic models, and the columns represent the number of sightings used to fit the models. The numbers in the right corner of each map represent the number of sightings used to fit the model. The scale is in individuals.km-2 (Ind/km^2^) for the NB-GAM, the TW-GAM and the ZIP-GAM and in the probability of presence (Pr.prob) for MaxEnt.
(TIFF)

S7 Fig Averaged uncertainty maps representing the coefficient of variation of each data-thinning rate in % associated with the averaged predictive density of dolphin group, from 25 to 75% of sighting thinning. The rows represent the different types of generic models, and the columns represent the number of sightings used to fit the models. The numbers in the right corner of each map represent the number of sightings used to fit the model. Due to very high isolated values, the maps were not contrasted so each coefficient of variation value beyond the 99% quantile were truncated. Dotted lines represent the survey area.
(TIFF)

S8 Fig Averaged uncertainty maps representing the coefficient of variation of each data-thinning rate in % associated with the averaged predictive density of dolphin group, from 90 to 95% of sighting thinning. The rows represent the different types of generic models, and the columns represent the number of sightings used to fit the models. The numbers in the right corner of each map represent the number of sightings used to fit the model. Due to very high isolated values, the maps were not contrasted so each coefficient of variation value beyond the 99% quantile were truncated. Dotted lines represent the survey area.
(TIFF)

S9 Fig Prediction maps of auks averaged over 100 models fitted to thinned datasets for each type of model from 25 to 90% of sighting thinning. The rows represent the different types of generic models, and the columns represent the number of sightings used to fit the models. The numbers in the right corner of each map represent the number of sightings used to fit the model. The scale is in individuals.km-2 (Ind/km^2^) for the NB-GAM, the TW-GAM and the ZIP-GAM and in the probability of presence (Pr.prob) for MaxEnt.
(TIFF)

S10 Fig Prediction maps of auks averaged over 100 models fitted to thinned datasets for each type of model from 92 to 99% of sighting thinning. The rows represent the different types of generic models, and the columns represent the number of sightings used to fit the models. The numbers in the right corner of each map represent the number of sightings used to fit the model. The scale is in individuals.km-2 (Ind/km^2^) for the NB-GAM, the TW-GAM and the ZIP-GAM and in the probability of presence (Pr.prob) for MaxEnt.
(TIFF)

S11 Fig Averaged uncertainty maps representing the coefficient of variation of each thinning rate in % associated with the averaged predictive density of auk group, from 25 to 90% of sighting thinning. The rows represent the different types of generic models, and the columns represent the number of sightings used to fit the models. The numbers in the right corner of each map represent the number of sightings used to fit the model. Due to very high isolated values, the maps were not contrasted so each coefficient of variation value beyond the 99% quantile were truncated. Dotted lines represent the survey area.
(TIFF)

S12 Fig Averaged uncertainty maps representing the coefficient of variation of each thinning rate in % associated with the averaged predictive density of auk group, from 92 to 99% of sighting thinning. The rows represent the different types of generic models, and the columns represent the number of sightings used to fit the models. The numbers in the right corner of each map represent the number of sightings used to fit the model. Due to very high isolated values, the maps were not contrasted so each coefficient of variation value beyond the 99% quantile were truncated. Dotted lines represent the survey area.
(TIFF)
