# Supplementary material for: How many sightings to model rare marine species distributions
Source: PLoS One. 2018 Mar 12;13(3):e0193231. doi: 10.1371/journal.pone.0193231 (PMC5846783; doi:10.1371/journal.pone.0193231)
Supplement: S1 Additional — (PDF) [file pone.0193231.s001.pdf]

Appendix 2: Some key concepts about the models used in the study.

- Negative Binomial distribution

The Negative Binomial distribution is extended from the Poisson regression and is defined by two parameters, the arithmetic mean and an exponent *k*. By modulating this exponent k, this distribution can be adapted to over-dispersed data (Bliss and Fisher, 2016). If the response variable Y obeys to a Negative Binomial distribution, the variance V(Y) and the mean E(Y) are related by the relationship $V(Y) = E(Y) + kE(Y)^2$ (Ver Hoef and Boveng, 2007).

In this study, we wanted to fit a GAM with a Negative Binomial distribution so we used the R package '`mgcv`' (Wood, 2006; 2013) and the `gam` function specifying the "Negative Binomial" family. Besides, we used the '`nb`' function to estimate the parameter *k* during the fitting.

- Tweedie distribution

The Tweedie distribution is useful to model continuous positive data because, compared to the Poisson distribution, it includes an additional parameter *p* which defines the model distribution. Indeed, if *p=0*, it is a normal distribution, if *p=1*, it is a Poisson distribution and if *p=2*, it is a Gamma distribution. Tweedie models can handle zero-inflated data (*i.e.* data with many zeros), because when $1<p<2$, they are a Poisson mixture of Gammas distributions (Arcuti et al., 2013). Besides, for a response variable Y that obeys a Tweedie distribution, the variance V(Y) and the mean E(Y) are related by the relationship $V(Y) = \varphi E(Y)^p$ where φ represents the dispersion parameter (Dunn and Smyth, 2005).

To fit a GAM with a Tweedie distribution, we used the R package '`mgcv`' (Wood, 2006; 2013) and the '`gam`' function specifying the "Tweedie" family. Besides, as we ignored the value of the parameter *p*, we used the '`tw`' function which estimates this parameter during the fitting.

- Zero-inflated Poisson distribution

Zero-inflated Poisson distribution is used to model count data with extra zero counts by modelling independently the count values, with a Poisson distribution (Zeileis et al., 2007), and the excess of zeros (Lambert, 1992). Thus, the ZIP regression is divided into two parts in which the species probability of presence and, given the presence, the species abundance are modelled sequentially (Ridout et al., 1998; Wenger and Freeman, 2014).

In the study, we tested the ZIP distribution with a GAM which showed nonlinear relationships between the response variable and the environmental predictors. To fit the model, we used the 'mgcv' package and the gam function but we specified the ZIP family and we used the 'ziP' function to estimate the θ parameter. This parameter includes two parameters which control the slope and the intercept of the zero model (Wood, 2006; 2013). To fit smooth functions for the GAM we introduced a *k* parameter which worth 4, for 4 degrees of freedom.

Arcuti, S. et al., 2013. Spatio-temporal modelling of zero-inflated deep-sea shrimp data by Tweedie generalized additive models. *Statistica, 73*(1), 87–101.

Bliss, C.I., Fisher, R.A., 2016. Fitting the Negative Binomial Distribution to Biological Data. *Biometrics, 9*(2), 176–200.

Shono, H., 2008. Application of the Tweedie distribution to zero-catch data in CPUE analysis. *Fisheries Research. 93*, 154–162.

Ver Hoef, J.M., Boveng, P.L., 2007. Quasi-poisson vs. negative binomial regression: How should we model overdispersed count data? *Ecology, 88*(11), 2766–2772.
