# Supplementary figures and images for: How many sightings to model rare marine species distributions
Source: PLoS One. 2018 Mar 12;13(3):e0193231. doi: 10.1371/journal.pone.0193231 (PMC5846783; doi:10.1371/journal.pone.0193231)

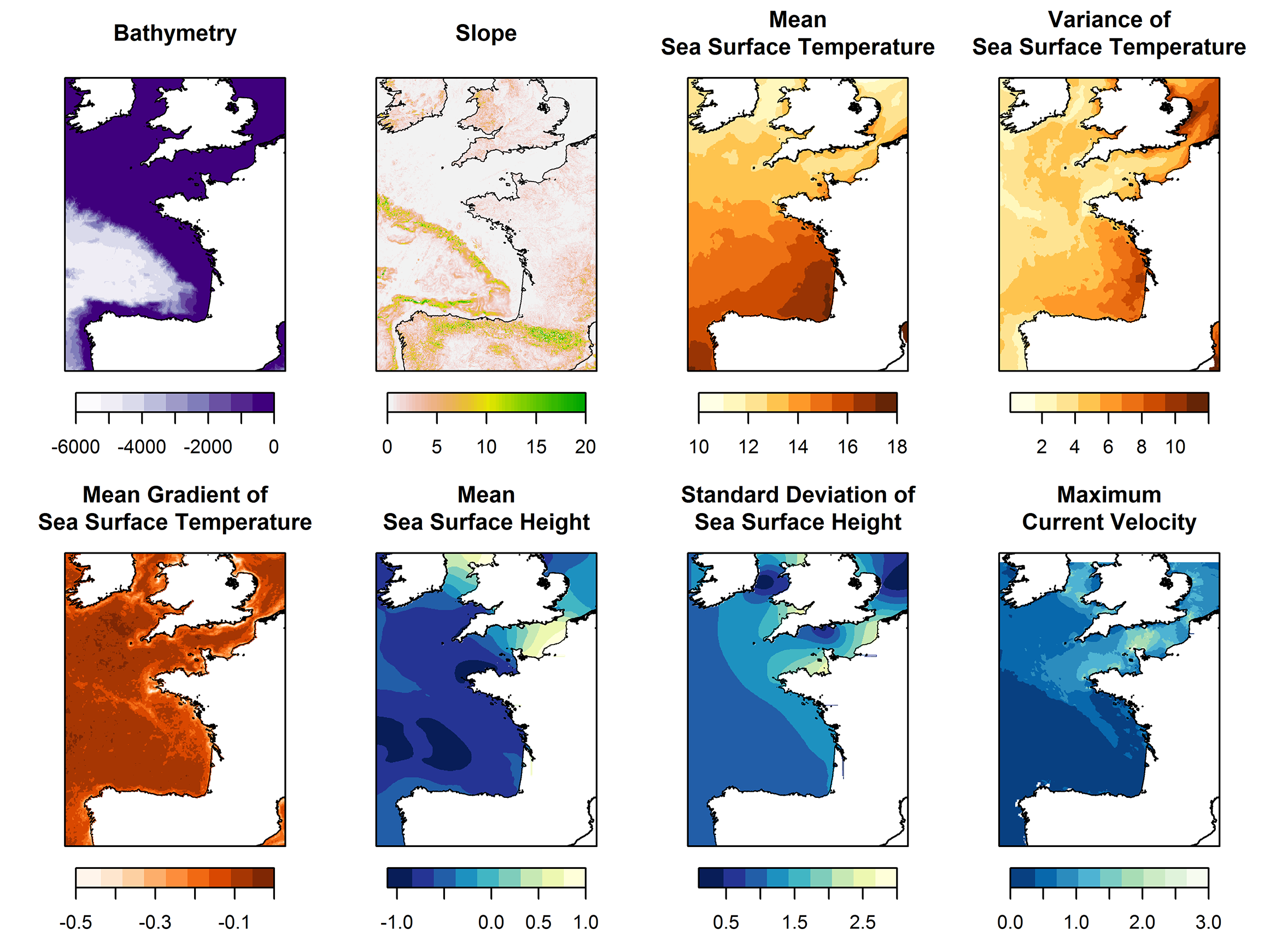

Supplement: S1 Fig — (TIF) [file pone.0193231.s002.tif]

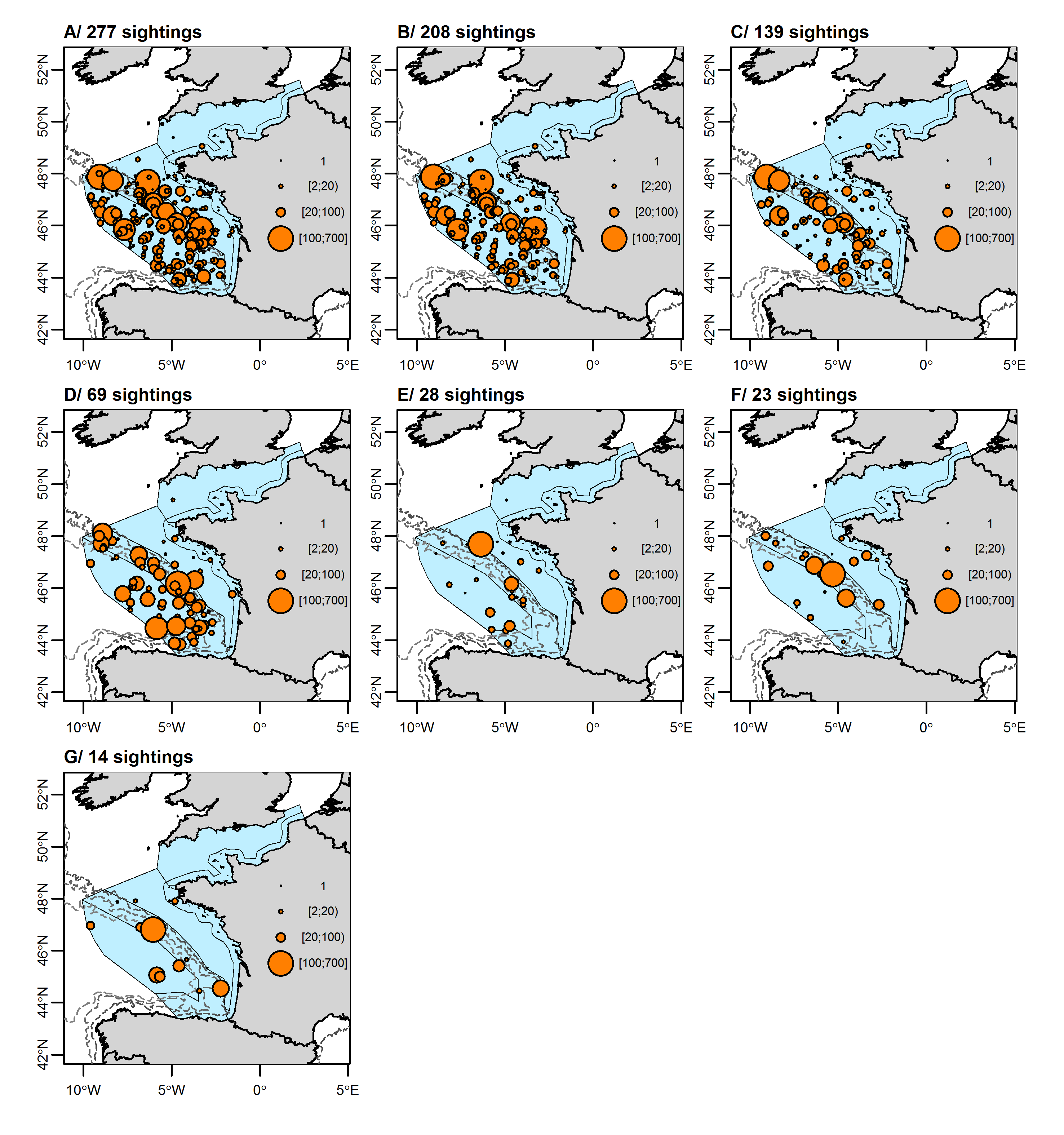

Supplement: S2 Fig — Sightings are classified by group sizes (1; 2–20; 20–100 and 100–700 individuals) with each point representing a group of individuals. (TIF) [file pone.0193231.s003.tif]

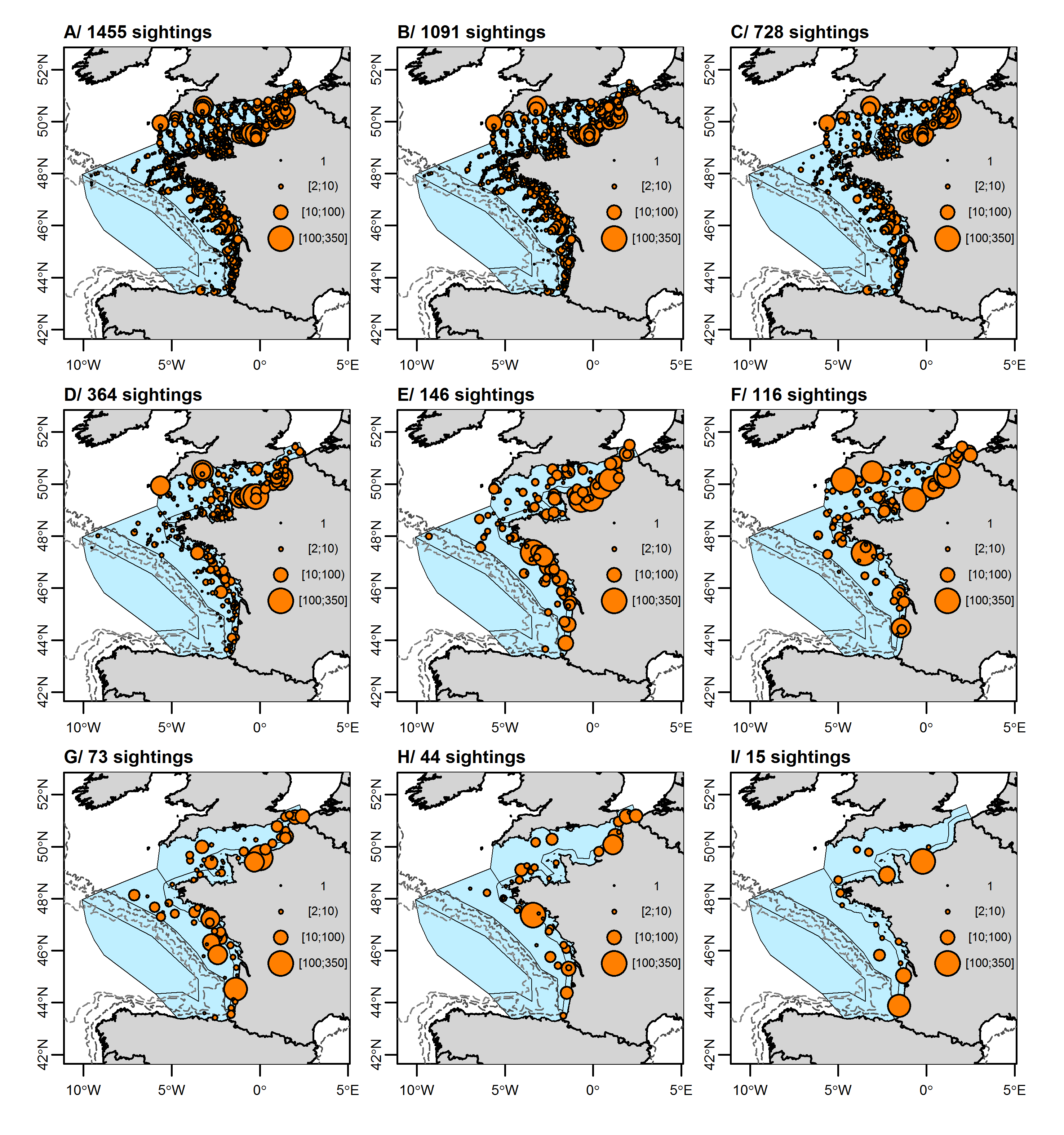

Supplement: S3 Fig — Sightings are classified by group sizes (1; 2–10; 10–100 and 100–350 individuals) with each point representing a group of individuals. (TIF) [file pone.0193231.s004.tif]

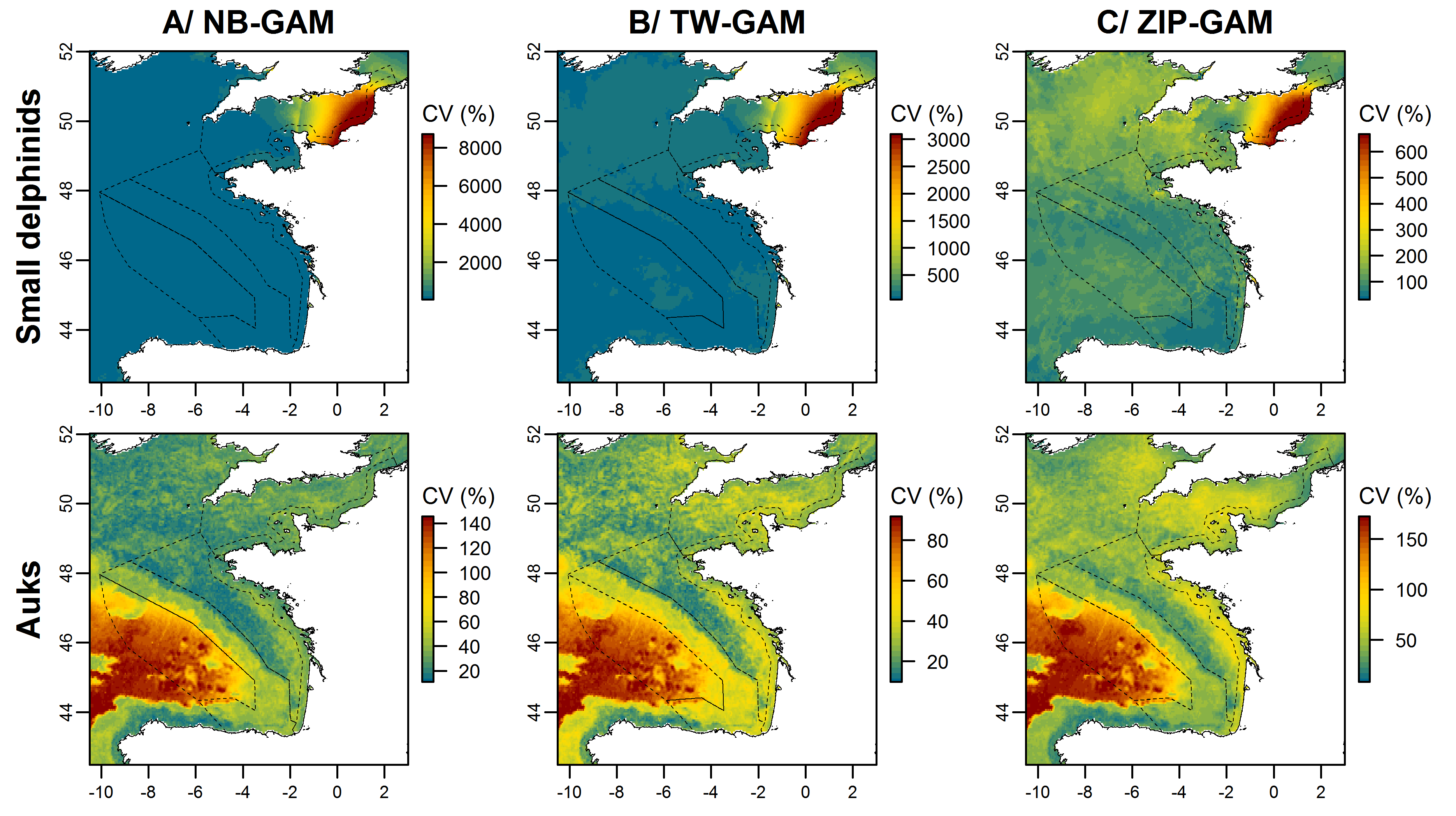

Supplement: S4 Fig — Uncertainty maps representing the coefficient of variation in % associated with the predictive relative density of dolphin and auk groups. Dotted lines represent the survey area. (TIFF) [file pone.0193231.s005.tiff]

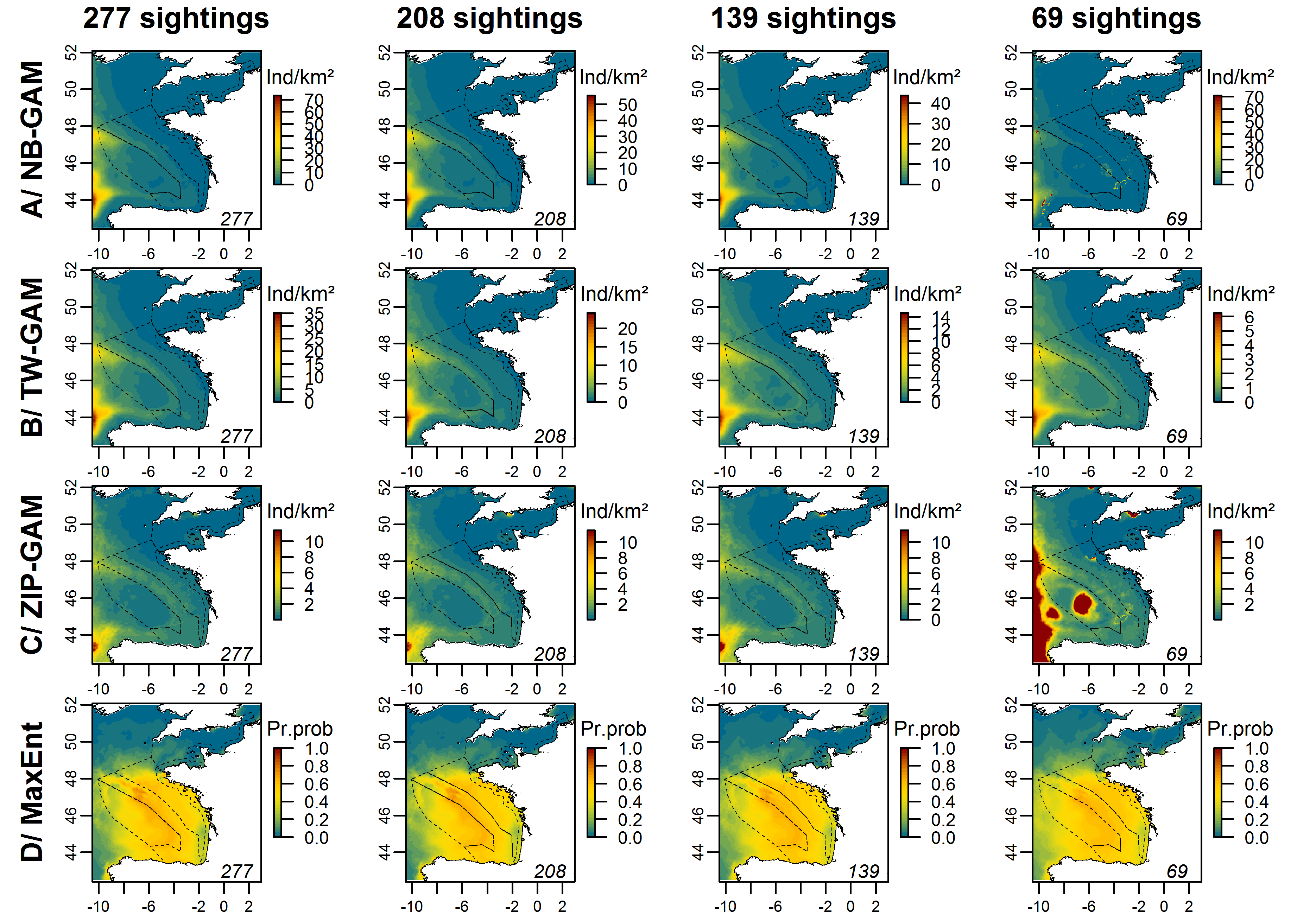

Supplement: S5 Fig — The rows represent the different types of generic models, and the columns represent the number of sightings used to fit the models. The numbers in the right corner of each map represent the number of sightings used to fit the model. The scale is in individuals.km-2 (Ind/km2) for the NB-GAM, the TW-GAM and the ZIP-GAM and in the probability of presence (Pr.prob) for MaxEnt. (TIFF) [file pone.0193231.s006.tiff]

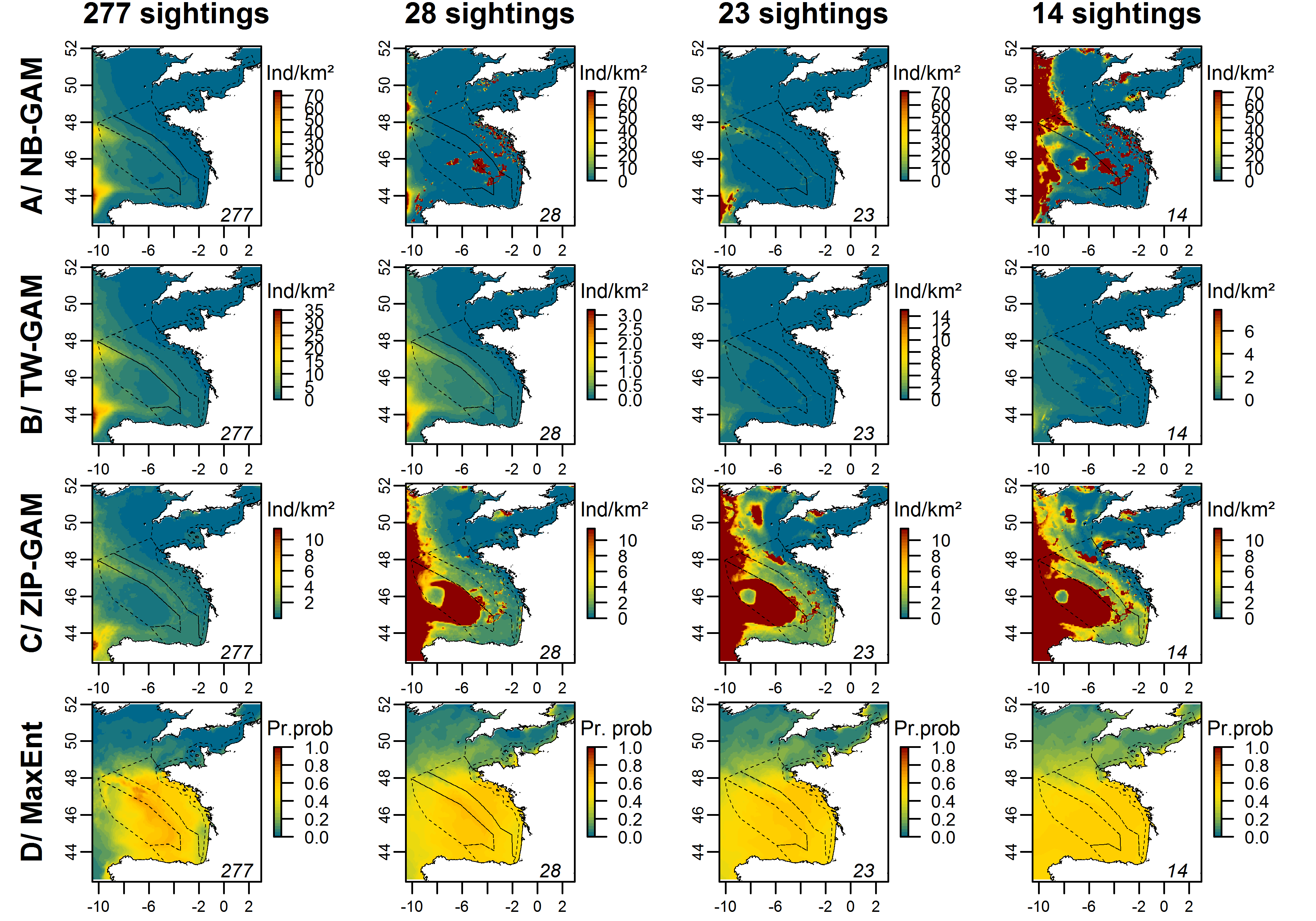

Supplement: S6 Fig — The rows represent the different types of generic models, and the columns represent the number of sightings used to fit the models. The numbers in the right corner of each map represent the number of sightings used to fit the model. The scale is in individuals.km-2 (Ind/km2) for the NB-GAM, the TW-GAM and the ZIP-GAM and in the probability of presence (Pr.prob) for MaxEnt. (TIFF) [file pone.0193231.s007.tiff]

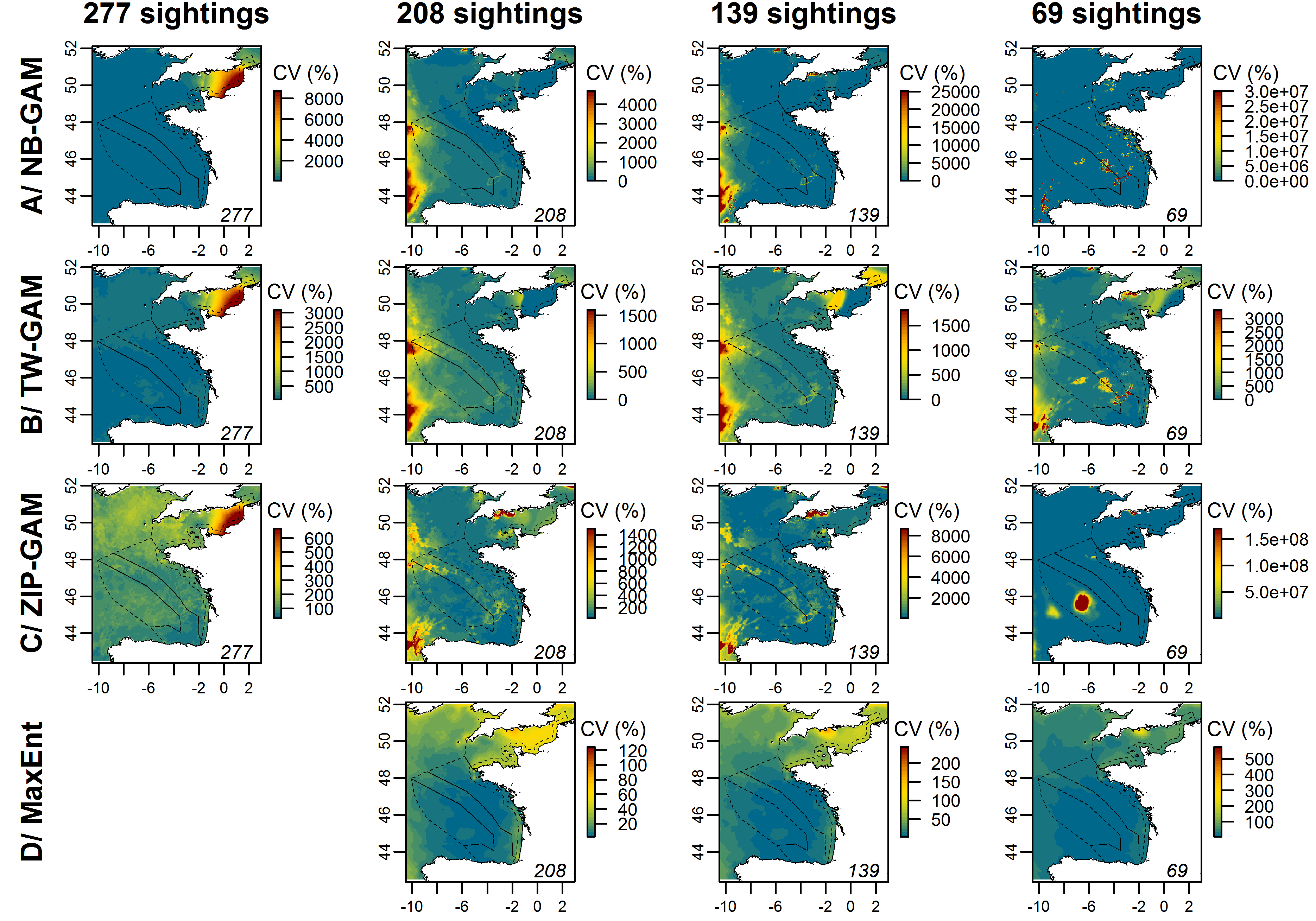

Supplement: S7 Fig — The rows represent the different types of generic models, and the columns represent the number of sightings used to fit the models. The numbers in the right corner of each map represent the number of sightings used to fit the model. Due to very high isolated values, the maps were not contrasted so each coefficient of variation value beyond the 99% quantile were truncated. Dotted lines represent the survey area. (TIFF) [file pone.0193231.s008.tiff]

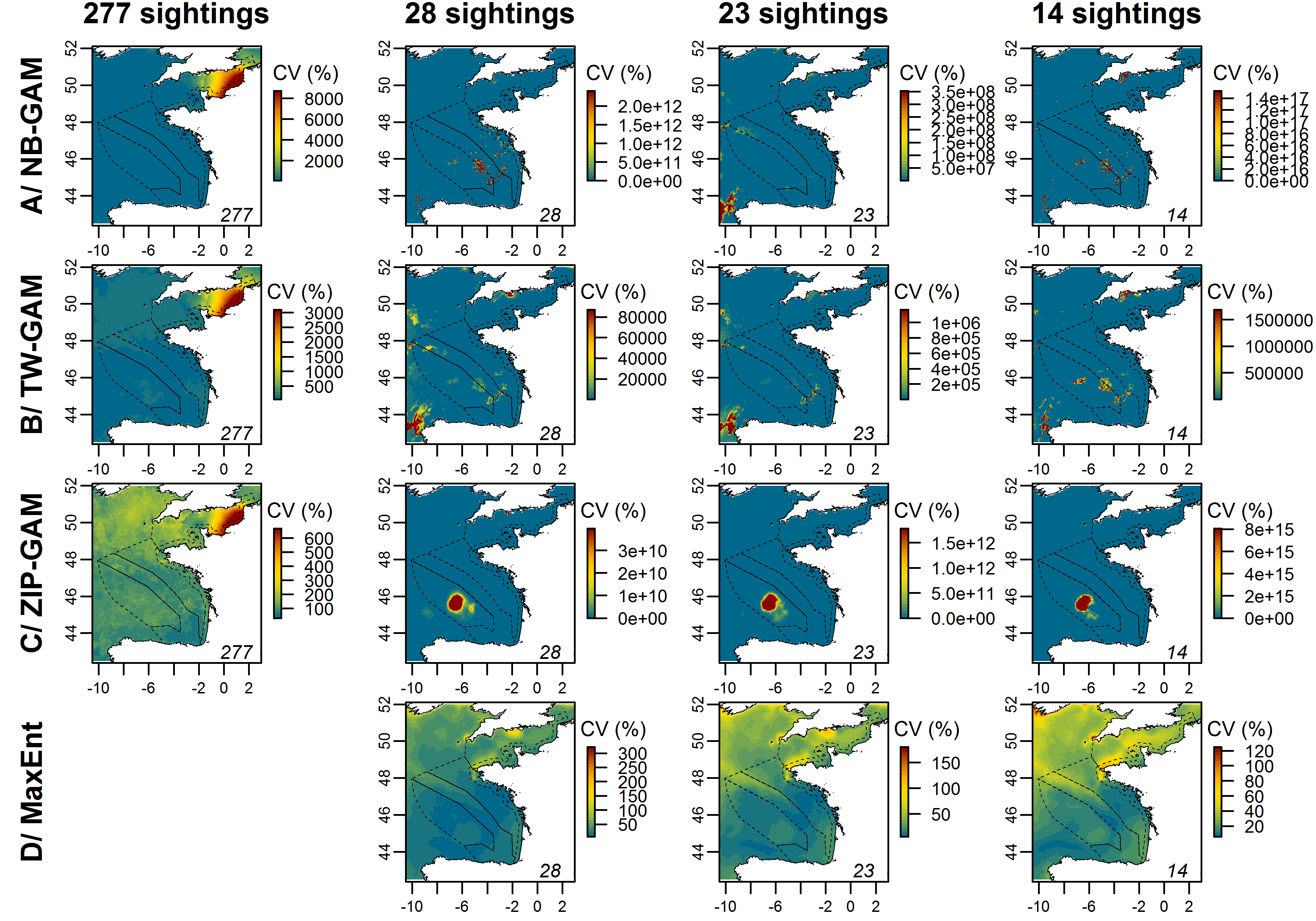

Supplement: S8 Fig — The rows represent the different types of generic models, and the columns represent the number of sightings used to fit the models. The numbers in the right corner of each map represent the number of sightings used to fit the model. Due to very high isolated values, the maps were not contrasted so each coefficient of variation value beyond the 99% quantile were truncated. Dotted lines represent the survey area. (TIFF) [file pone.0193231.s009.tiff]

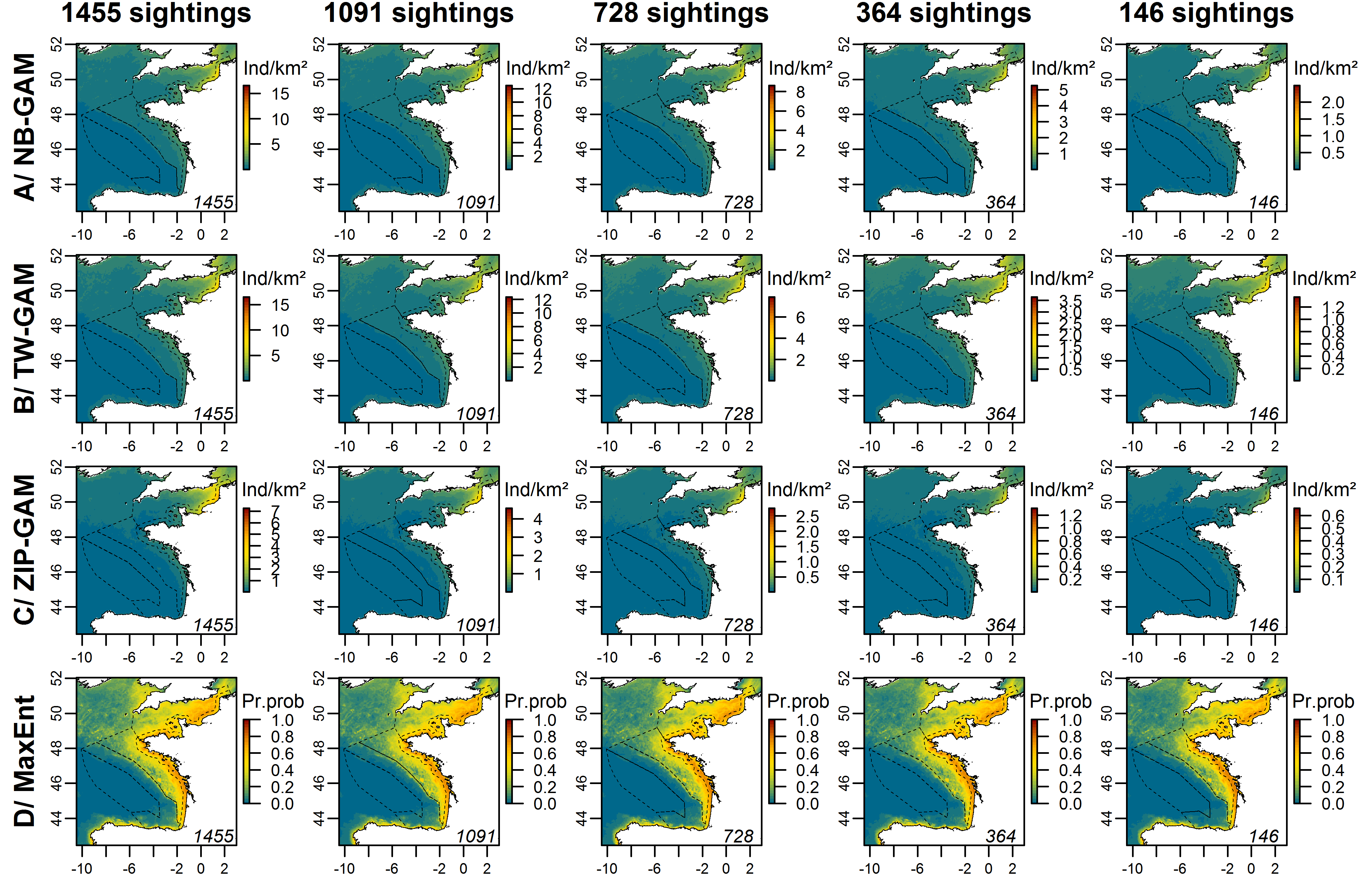

Supplement: S9 Fig — The rows represent the different types of generic models, and the columns represent the number of sightings used to fit the models. The numbers in the right corner of each map represent the number of sightings used to fit the model. The scale is in individuals.km-2 (Ind/km2) for the NB-GAM, the TW-GAM and the ZIP-GAM and in the probability of presence (Pr.prob) for MaxEnt. (TIFF) [file pone.0193231.s010.tiff]

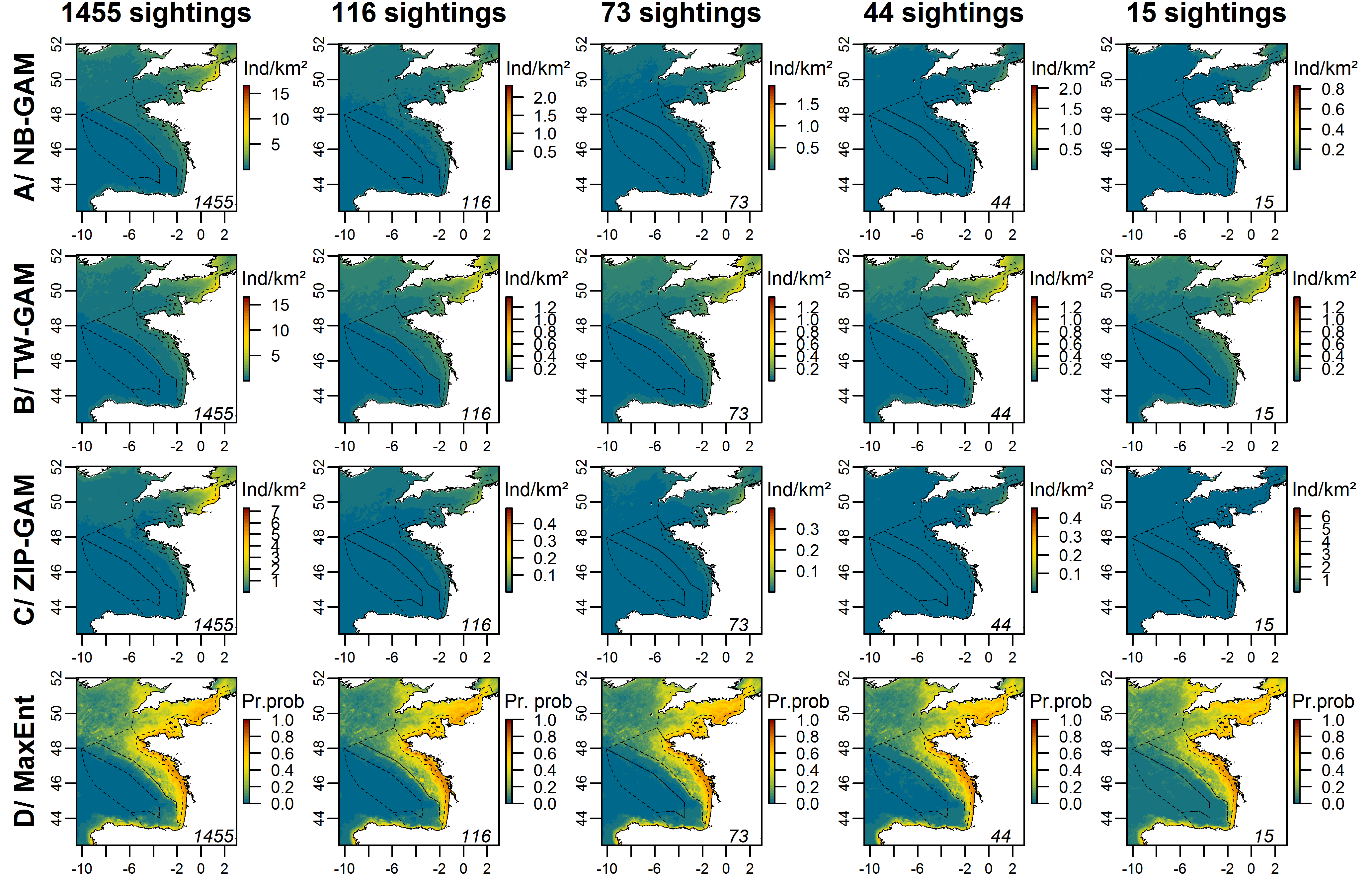

Supplement: S10 Fig — The rows represent the different types of generic models, and the columns represent the number of sightings used to fit the models. The numbers in the right corner of each map represent the number of sightings used to fit the model. The scale is in individuals.km-2 (Ind/km2) for the NB-GAM, the TW-GAM and the ZIP-GAM and in the probability of presence (Pr.prob) for MaxEnt. (TIFF) [file pone.0193231.s011.tiff]

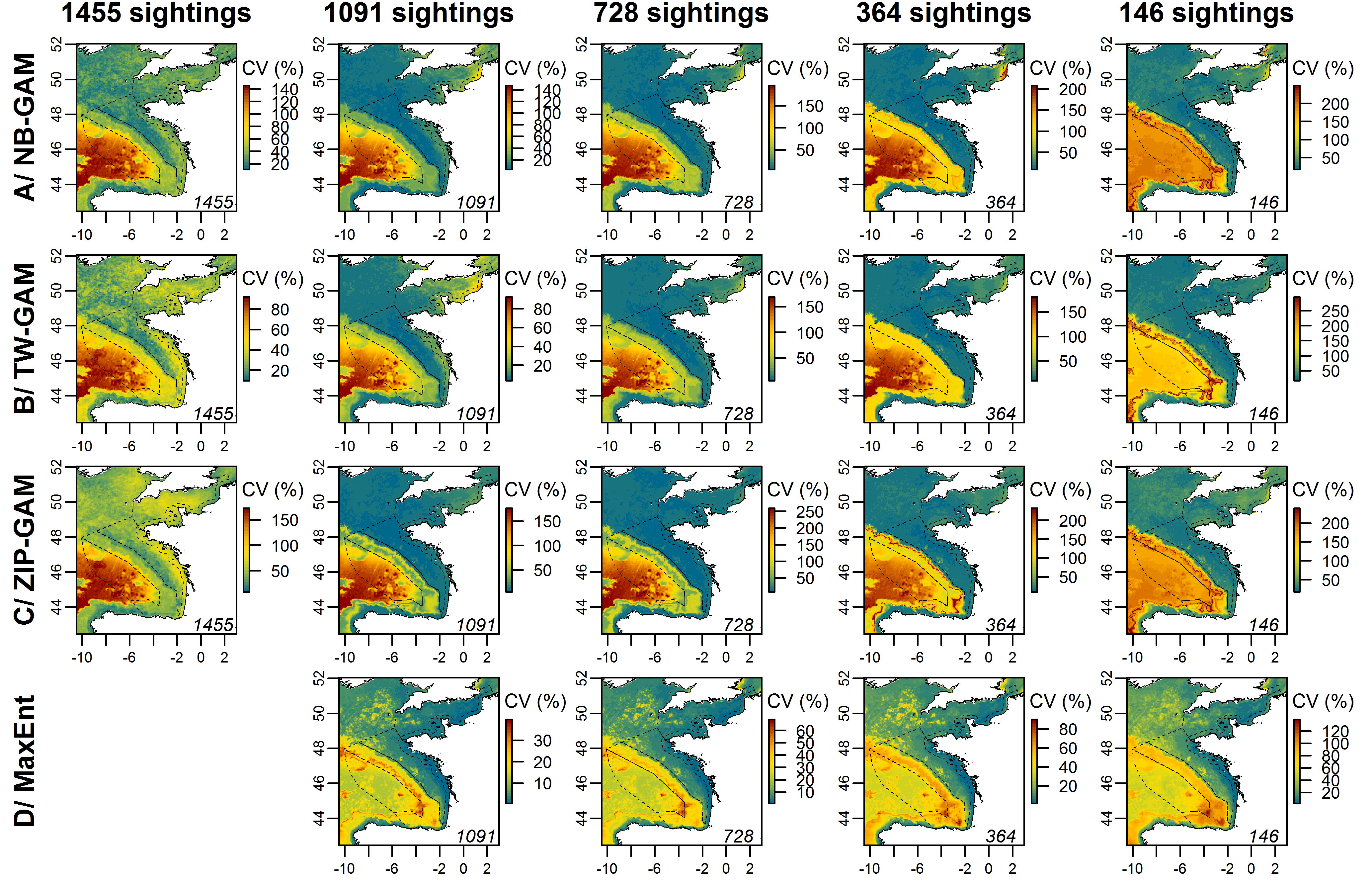

Supplement: S11 Fig — The rows represent the different types of generic models, and the columns represent the number of sightings used to fit the models. The numbers in the right corner of each map represent the number of sightings used to fit the model. Due to very high isolated values, the maps were not contrasted so each coefficient of variation value beyond the 99% quantile were truncated. Dotted lines represent the survey area. (TIFF) [file pone.0193231.s012.tiff]

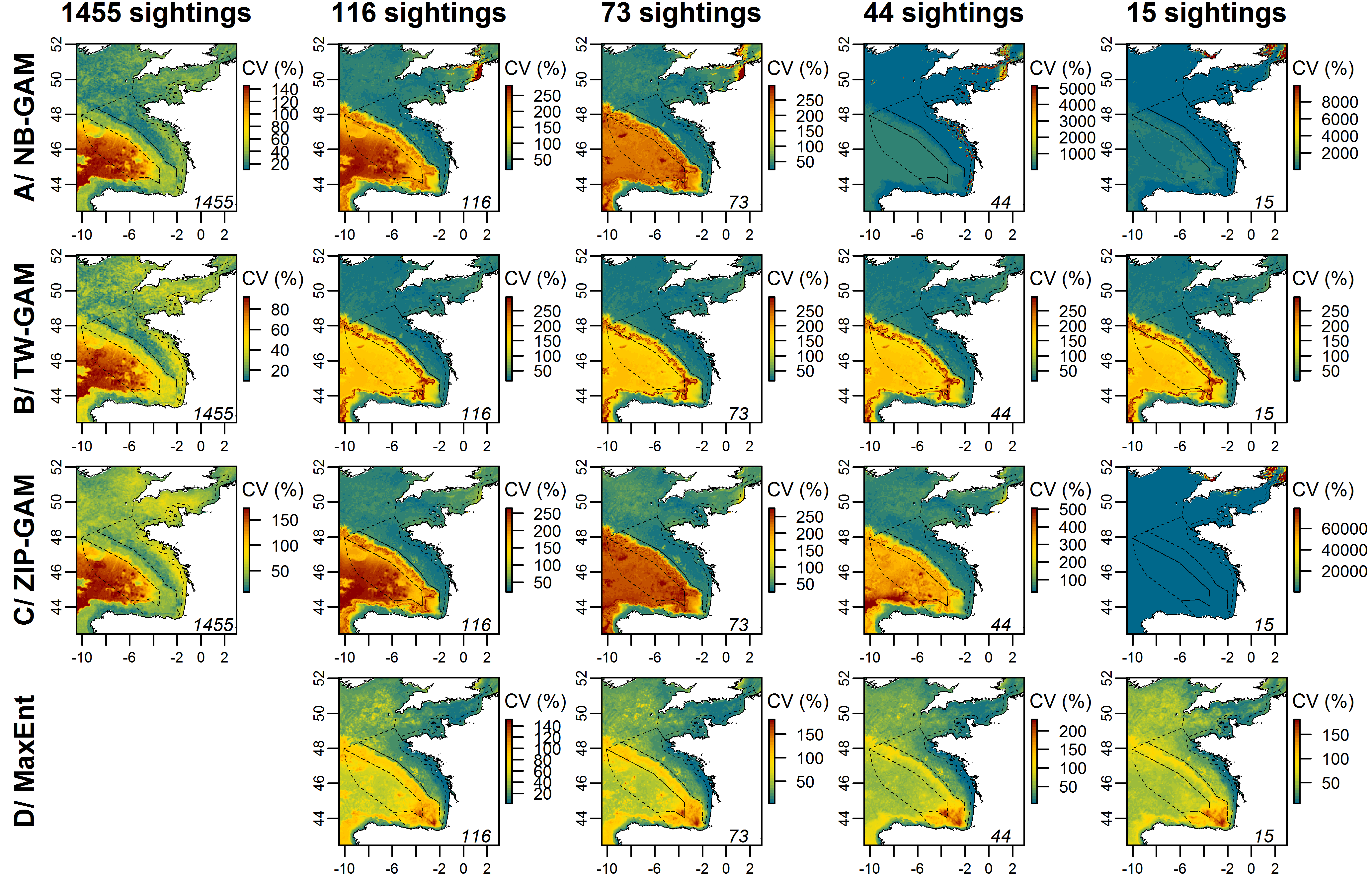

Supplement: S12 Fig — The rows represent the different types of generic models, and the columns represent the number of sightings used to fit the models. The numbers in the right corner of each map represent the number of sightings used to fit the model. Due to very high isolated values, the maps were not contrasted so each coefficient of variation value beyond the 99% quantile were truncated. Dotted lines represent the survey area. (TIFF) [file pone.0193231.s013.tiff]
